# The anti-sigma factor MucA of *Pseudomonas aeruginosa*: Dramatic differences of a *mucA22* vs. a Δ*mucA* mutant in anaerobic acidified nitrite sensitivity of planktonic and biofilm bacteria *in vitro* and during chronic murine lung infection

**DOI:** 10.1371/journal.pone.0216401

**Published:** 2019-06-03

**Authors:** Warunya Panmanee, Shengchang Su, Michael J. Schurr, Gee W. Lau, Xiaoting Zhu, Zhaowei Ren, Cameron T. McDaniel, Long J. Lu, Dennis E. Ohman, Daniel A. Muruve, Ralph J. Panos, Hongwei D. Yu, Thomas B. Thompson, Boo Shan Tseng, Daniel J. Hassett

**Affiliations:** 1 Department of Molecular Genetics, Biochemistry and Microbiology, University of Cincinnati College of Medicine, Cincinnati, OH United States of America; 2 Department of Immunology and Microbiology, University of Colorado School of Medicine, Aurora, CO United States of America; 3 College of Veterinary Medicine, University of Illinois at Urbana-Champaign, Urbana, IL United States of America; 4 Division of Biomedical Informatics, Cincinnati Children’s Hospital Medical Center, Cincinnati, OH United States of America; 5 Department of Microbiology and Immunology, Virginia Commonwealth University Medical Center, Richmond, VA United States of America; 6 McGuire Veterans Affairs Medical Center, Richmond, VA United States of America; 7 Department of Medicine, University of Calgary, Calgary, Alberta, Canada; 8 Department of Medicine, Cincinnati Veterans Affairs Medical Center, Cincinnati, OH United States of America; 9 Pulmonary, Critical Care, and Sleep Division, Department of Medicine, University of Cincinnati College of Medicine, Cincinnati, OH United States of America; 10 Department of Biochemistry and Microbiology, Marshall University, Huntington, WV United States of America; 11 Department of Life Sciences, University of Nevada-Las Vegas, Las Vegas, NV United States of America; East Carolina University Brody School of Medicine, UNITED STATES

## Abstract

Mucoid *mucA22 Pseudomonas aeruginosa* (*PA*) is an opportunistic lung pathogen of cystic fibrosis (CF) and chronic obstructive pulmonary disease (COPD) patients that is highly sensitive to acidified nitrite (A-NO_2_^-^). In this study, we first screened *PA* mutant strains for sensitivity or resistance to 20 mM A-NO_2_^-^ under anaerobic conditions that represent the chronic stages of the aforementioned diseases. Mutants found to be sensitive to A-NO_2_^-^ included PA0964 (*pmpR*, PQS biosynthesis), PA4455 (probable ABC transporter permease), *katA* (major catalase, KatA) and *rhlR* (quorum sensing regulator). In contrast, mutants lacking PA0450 (a putative phosphate transporter) and PA1505 (*moaA2*) were A-NO_2_^-^ resistant. However, we were puzzled when we discovered that *mucA22* mutant bacteria, a frequently isolated *mucA* allele in CF and to a lesser extent COPD, were more sensitive to A-NO_2_^-^ than a truncated **Δ***mucA* deletion (**Δ**157–194) mutant in planktonic and biofilm culture, as well as during a chronic murine lung infection. Subsequent transcriptional profiling of anaerobic, A-NO_2_^-^-treated bacteria revealed restoration of near wild-type transcript levels of protective NO_2_^-^ and nitric oxide (NO) reductase (*nirS* and *norCB*, respectively) in the Δ*mucA* mutant in contrast to extremely low levels in the A-NO_2_^-^-sensitive *mucA22* mutant. Proteins that were *S*-nitrosylated by NO derived from A-NO_2_^-^ reduction in the sensitive *mucA22* strain were those involved in anaerobic respiration (NirQ, NirS), pyruvate fermentation (UspK), global gene regulation (Vfr), the TCA cycle (succinate dehydrogenase, SdhB) and several double mutants were even more sensitive to A-NO_2_^-^. Bioinformatic-based data point to future studies designed to elucidate potential cellular binding partners for MucA and MucA22. Given that A-NO_2_^-^ is a potentially viable treatment strategy to combat PA and other infections, this study offers novel developments as to how clinicians might better treat problematic PA infections in COPD and CF airway diseases.

## Introduction

Chronic obstructive pulmonary disease (COPD) and cystic fibrosis (CF) are two chronic airway diseases complicated by life-threatening infections caused by the opportunistic Gram-negative bacterium, *Pseudomonas aeruginosa (PA)*. In the United States alone, there are an estimated 15 million COPD patients (251 million worldwide (WHO estimates, [1]), while there are only 30,000 U.S. CF patients (70,000 world-wide, [2]). COPD is a highly deteriorative alveolar disease coupled with airway derangements causing an accumulation of thick mucus that is typically a consequence of long-term smoking. Cultures of respiratory secretions yield *PA* in 8.5–16.8% of patients who also experience more frequent COPD exacerbations [3, 4] and up to 18% of patients requiring mechanical ventilation [5] who have increased mortality. In contrast, CF airway disease is biphasic, involving an early oxidative, neutrophil-rich environment [6] (especially during acute exacerbations) [7] followed by a chronic hypoxic or anaerobic phase [8–10]. In contrast, chronically infected patients suffer from poor airway oxygenation resulting, in part, from intractable, antibiotic-resistant [11] bacterial communities known as biofilms that are formed by *PA* and other organisms embedded within thick microaerobic [12] or anaerobic airway mucus [8, 13–16].

A further reduction in lung function occurs when mucoid, *mucA22* mutant alginate-overproducing mutant *PA* forms biofilms that are of the mode II variety [13, 17, 18]. The *mucA22* allele results from a C residue deletion at position 430, leaving a truncated 15.8 kDa MucA protein [19]. Unlike mode I biofilms, where organisms attach directly to animate (e.g., cells) or inanimate (e.g., plastic, glass, etc.) surfaces, mode II biofilm bacteria are not attached to surfaces, but rather to themselves, developing as highly structured communities embedded within the thick mucus that mature into “soccer ball-shaped” micro- and/or macrocolonies in the infected airways [20]. Many research groups around the world are attempting to unravel the precise metabolic features of *PA* within mode II biofilms embedded within the CF and COPD airway mucus [17, 21, 22]. Metabolism of certain amino acids appears to be prevalent among *PA* isolates from chronically infected patients including aromatic amino acids, specifically phenylalanine and tyrosine [23]. Two seminal papers were published in 2002 by our group (Yoon et al., [8]) and Worlitzsch et al., [13] that indicate that oxygen tension within the thick mucus lining of the CF airways is significantly reduced and there are some niches that are likely completely anaerobic. For many CF and COPD pathogens, including *PA*, an alternative electron acceptor such as nitrate (NO_3_^-^) or nitrite (NO_2_^-^) is required. Both NO_3_^-^ and NO_2_^-^ can be detected in sufficient quantities for *PA* to undergo anaerobic respiration in both CF [24] and COPD [25]. Since our anaerobic biofilm theory of chronic CF lung disease was reported in 2002 [8], obligate anaerobes have been isolated from CF sputum (for recent review, see [18]) as well as in COPD sputum [26]. In fact, there have been many published manuscripts spanning over 40 years describing the isolation of obligate anaerobes from the CF airways, thereby supporting our anaerobic theory [10, 27, 28] (for mini-reviews, see [17, 18]). In conjunction with anaerobic nitrogen oxide metabolism, one very significant development in the potential treatment of mucoid *PA* was discovered in 2006 where we (Yoon et al., [9]) also showed that mucoid *mucA22* mutant CF isolates were sensitive to acidified sodium nitrite (herein termed A-NO_2_^-^). A-NO_2_^-^ was used in the aforementioned study because we found that the pH of the CF airway surface liquid from explanted CF lungs was ~6.4–6.5 [9]. However, in that study, of nearly 100 mucoid *PA* isolates from 12 different North American CF clinics, expectedly none possessed true deletions of the *mucA* gene [9]. A second study, using the other two major COPD and CF pathogens methicillin-resistant *Staphylococcus aureus* and *Burkholderia cepacia*, as well as nonmucoid *PA* showed that A-NO_2_^-^ also kills these organisms, particularly under anaerobic conditions [29]. Relatedly, it should be noted that one feature of CF is reduced airway iNOS (**i**nducible nitric oxide (**NO**) **s**ynthase) expression, especially in chronic CF [30]. iNOS is an enzyme that generates potentially antimicrobial levels of nitric oxide (NO) and is a major contributor to the hosts innate immune system. In contrast, iNOS also appears to play a role in COPD, as iNOS mRNA levels were recently shown to be elevated in COPD patient’s relative to nonsmokers and smokers without COPD [31].

There is a severe dearth of nearly any formal understanding of the genetics of adaptive mutations in *PA* that are acquired in COPD. Puzzlingly, given the estimated 3,600-fold reduced patient numbers world-wide for CF relative to COPD, vastly more is understood regarding mutations that emerge during the course of CF [32, 33]. For example, *PA* acquires 3 adaptive mutations during CF, *lasR* (early $\bar{x}$ = 12 years, *rhlR* ($\bar{x}$ = 17 years [34]) and *mucA* (early [in some cases 3 yrs old] and late (chronic) CF [9, 35] and likely also in COPD [36]. In 2002, we discovered yet another weakness in mutants frequently isolated from CF patient sputum. We discovered that anaerobic *PA rhlR* QS (controlled by the *las* system) mutants in *PA* anaerobic biofilms mysteriously committed an anaerobic metabolic suicide in biofilms by overproduction of toxic endogenous levels of respiratory NO [8]. Chronic, long-term infections are characterized by bacteria that have undergone a process known as mucoid conversion within the progressively thickened airway mucus [37]. This process involves mutations in a variety of genes including *mucB (algN)* (periplasmic protein that binds the anti-sigma factor, MucA, [38, 39]), *algW* (encoding a membrane protease that cleaves MucA [40]), and *mucD* (a periplasmic protease that degrades MucA via activation of MucP [39, 41]. However, the most abundant mutations that trigger mucoid conversion in both COPD [31, 36] and CF [9, 35] are within the *mucA* gene, encoding a cytoplasmic membrane-spanning anti-sigma factor [35]. The primary appreciated function of MucA is to sequester the extracytoplasmic sigma factor AlgT(U) near the cytoplasmic side of the inner membrane [42]. The most common *mucA* mutant allele is called *mucA22* [9, 35], caused by a C deletion at base 430, resulting in a 15.8 kDa truncated protein that allows mucoid conversion by enabling AlgT(U) to activate transcription of genes involved in production of the viscous exopolysaccharide alginate, a large linear *β*-1,4-linked co-polymer consisting of *β*-D-mannuronate and *α*-L-guluronate [43, 44]. The production of alginate severely complicates the clinical course for CF patients [45], resulting in progressively worsened forced expiratory volume per second (FEV_1_) measurements and poor pulmonary function tests (PFTs). Thus, mucoid conversion is often considered one, if not the most negative, clinical hallmarks precipitating a dramatic antibiotic regimen adjustment for patients infected by such organisms.

Given this important and comprehensive background information, in this study, we elected to first identify a series of transposon mutants that were either more susceptible or resistant to defined concentrations of anaerobic A-NO_2_^-^. During this process, we discovered a different and unexpected role of MucA in sensitivity to A-NO_2_^-^ by generating not only *mucA22* mutants, but also a truncated Δ*mucA* mutant (**Δ**157–194), the latter of which were surprisingly resistant to A-NO_2_^-^ relative to its *mucA22* counterpart. We used a combined transcriptional profiling and protein *S*-nitrosylation approach to identify potential mechanisms of A-NO_2_^-^ sensitivity in wild-type, *mucA22* vs. Δ*mucA* strains. First, the transcription levels of genes encoding **n**itr**a**te (NO_3_^-^), **ni**trite (NO_2_^-^) and **n**itric **o**xide (NO) **r**eductase (collectively NAR, NIR and NOR) were at or near wild-type levels in **Δ***mucA* and wild-type bacteria when compared to the *mucA22* mutant, which we show to be far lower in this study upon exposure to A-NO_2_^-^. Consistent with these observations, we show that *mucA22* bacteria are susceptible to A-NO_2_^-^ during a chronic lung infection in mice, while Δ*mucA* bacteria were resistant. Our data also involved extensive bioinformatics analysis suggesting that MucA22, a truncated ~15.8 kDa protein, has an as yet unknown anaerobic function, but also confers a significant defect, a translationally-significant and marked sensitivity to A-NO_2_^-^.

## Results

### Screening for *PA* strains that demonstrate enhanced sensitivity or resistance to A-NO_2_^-^

A mariner Tn library representing a three genome coverage (~15,000 mutants) and previously constructed insertion or deletion mutants were used to screen for *PA* strains for sensitivity or resistance to 20 mM A-NO_2_^-^ under anaerobic conditions (Table 1). Using wild-type PAO1 bacteria and strain FRD1 as respective A-NO_2_^-^ resistant and sensitive controls, several Tn mutants that were identified to be more resistant to A-NO_2_^-^. These included PA1504 (transcriptional regulator), PA0450 (probable PO_4_^3-^ transporter), PA1370 (hypothetical protein), and PA0780 (proline utilization regulator). In contrast, A-NO_2_^-^ sensitive strains included mutants lacking PA0964 (*pmpR*, regulator of PqsR-mediated quorum sensing, [46]), PA4455 (a putative ABC transporter permease [47]), ribonucleotide diphosphate reductase subunits (PA5496-*nrdJa*, 5497-*nrdJb*), *mucA22*, PA0779 (ATP-dependent protease), *katA* (major catalase KatA, [48]), *rhlR* (quorum sensing regulator, [49]), *lon* (Lon protease), *nuoK* (NADH dehydrogenase subunit), *norCB* (NO reductase, [50], clinical strain FRD1 and a proven A-NO_2_^-^ sensitive *mucA22* mutant [51], respectively. As a control, we constructed a deletion mutant of *mucA* (**Δ***mucA*) and to our surprise, this strain was resistant to A-NO_2_^-^.

**Table 1:** Transposon (Tn) and gene replacement mutants found to be sensitive (S) or resistant (R) to 20 mM A-NO_2_^-^ under anaerobic conditions after 24 hr incubation.

Strain Gene	(Sensitive (S)/Resistant (R)	Reference
FRD1 *mucA22*	S	[51]
PAO1 *mucA22*	S	[9], This study
PAO1 *pmpR*::*Tn-*Gm	S	This study
PAO1 *norCB*::Gm	S	[50]
PAO1 *rhlR*::Gm	S	[52]
PAO1 *katA*::Gm	S	[53]
PAO1 *lon*::*Tn-*Gm	S	This study
PAO1 *nrdJa*,*b*::*Tn-*Gm	S	This study
PAO1 *nuoK*::*Tn-*Gm	S	This study
PAO1 PA4455::Gm	S	[47], This study
PAO1 **Δ***mucA* (Δ157–194) (Hassett lab)	S	This study
PAO1 **Δ***mucA* (Δ157–194) (Schurr lab)	R	This study
PAO1 PA1504::*Tn-*Gm	R	This study
PAO1 PA0450::*Tn-*Gm	R	This study
PAO1 PA1370::*Tn-*Gm	R	This study
PAO1 PA0780::*Tn-*Gm	R	This study
PAO1 PA0780::*Tn-*Gm	R	This study

### The *mucA* gene, mucoidy and alginate production in *mucA22* vs. Δ*mucA* strains

**Fig 1:**
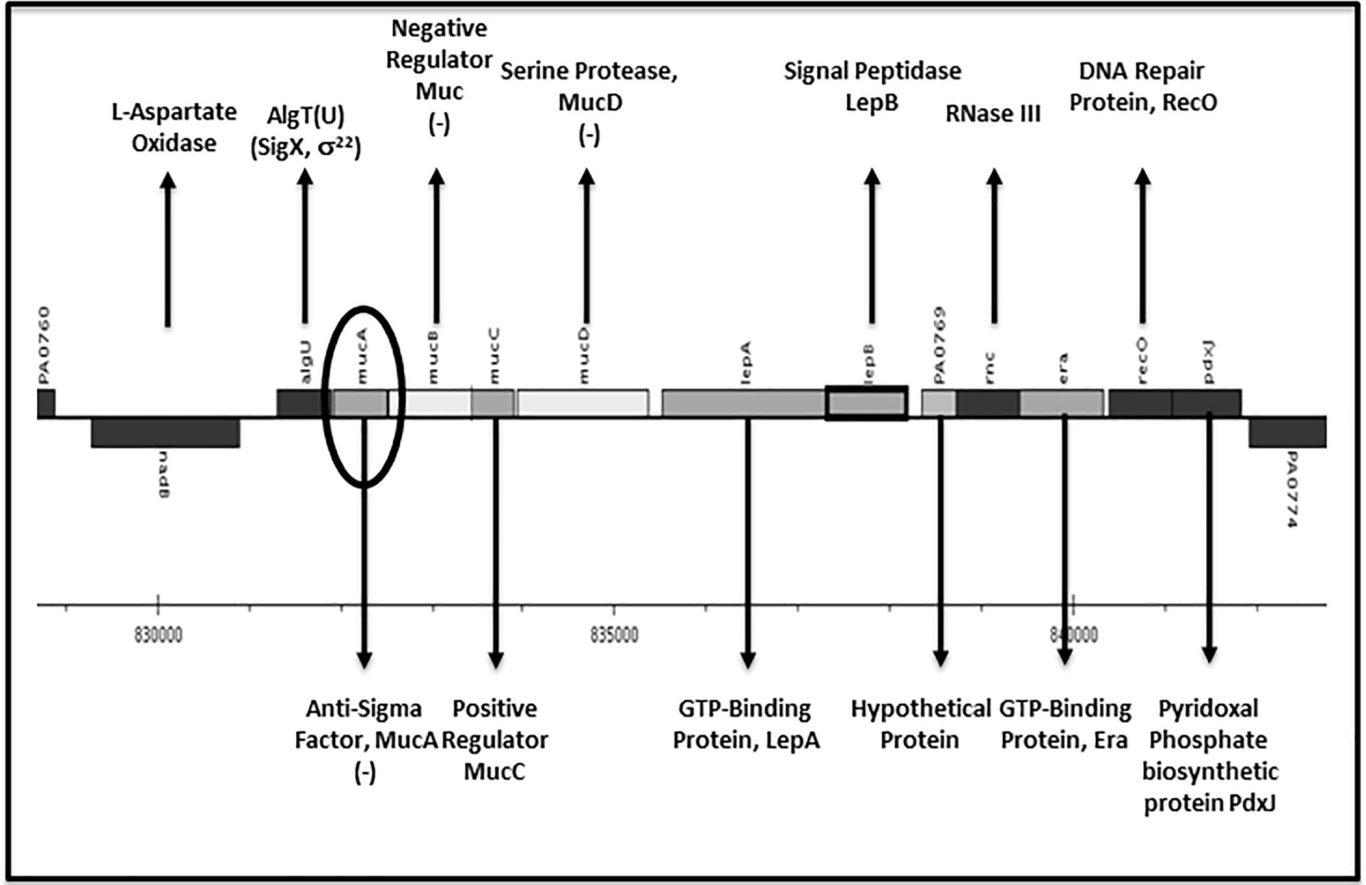
Genetic map and function of the gene products of >12 kb genomic region containing the *algT(U)mucABCD* genes (scanned from www.pseudomonas.com). The *mucA* gene immediately downstream of the *algT(U)* locus is circled. Minus signs (-) indicated genes involved in mucoid conversion when mutated [35, 39, 54, 55].

The immediate goal of our experimental plan moving forward was to assess the evolutionary “rationale” underlying why the *mucA22* mutation occurs so frequently in *PA* derived from sputum from patients suffering from both CF and COPD airway disease [9, 36] and evaluate whether a complete deletion of the *mucA* gene differed with respect to alginate production and sensitivity to the potential translational CF and COPD therapeutic agent, A-NO_2_^-^. As a reminder, the *mucA* gene encodes a cytoplasmic membrane spanning anti-sigma factor that binds the extra-cytoplasmic sigma factor AlgT(U) in the cytoplasm and is one of the most common mechanisms for the conversion to mucoidy in both of the aforementioned diseases [35]. The *mucA* gene is the second gene of an 11.524 kb alginate gene regulation operon located between 831,301 and 842,825 bp on the *PA* chromosome (Fig 1, www.pseudomonas.com). Other genes indicated by a minus sign (-) allow for mucoid conversion or alginate gene regulation when mutated (e.g., negative regulators *mucB*,*C*,*D*, etc. [38, 39, 54].

**Fig 2:**
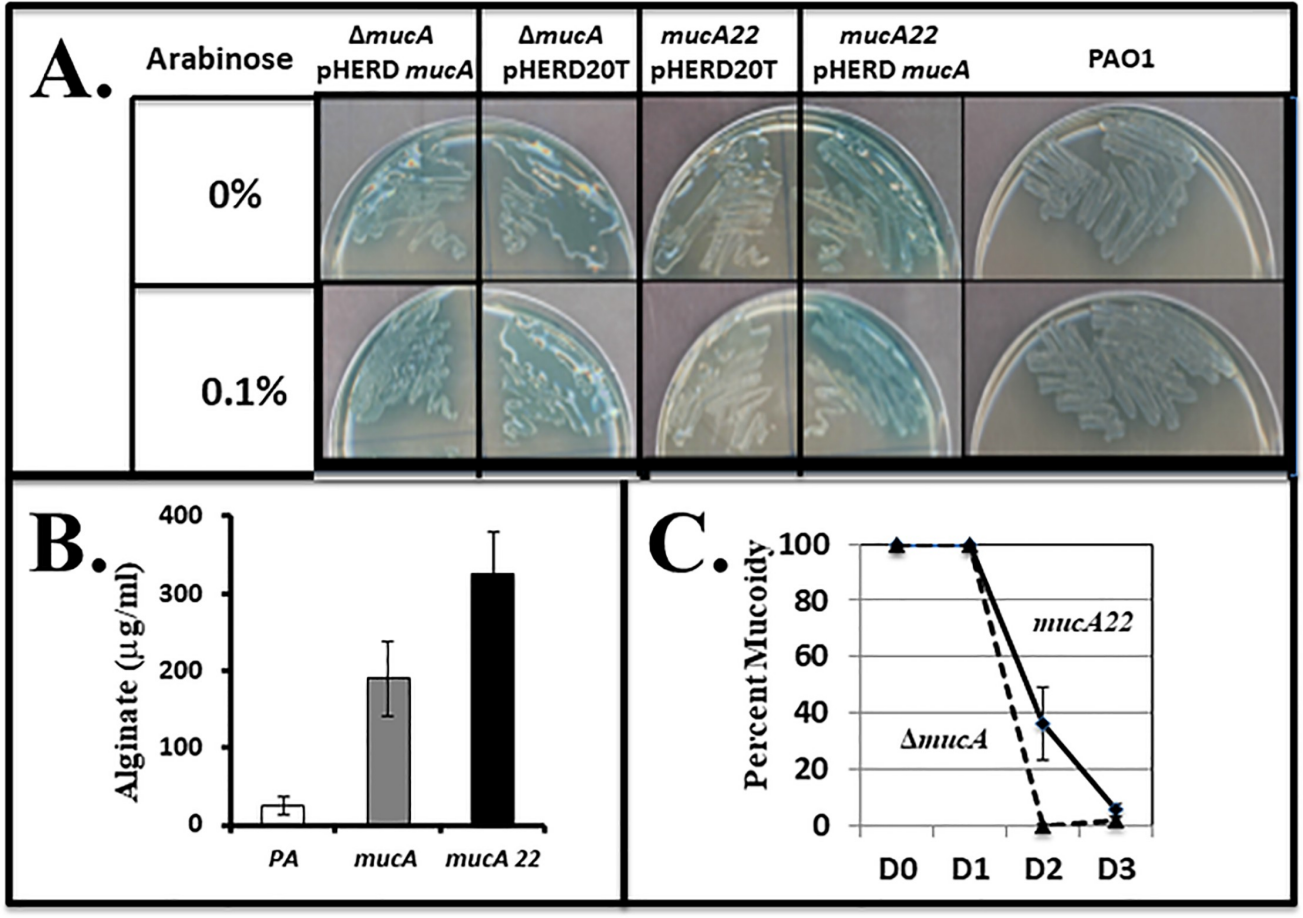
Differences in the mucoid phenotype of the *mucA22* and Δ*mucA* mutants. **A.** Colony morphology of wild-type, *mucA22*, and **Δ***mucA* bacteria on L-agar plates containing +/- of 0.1% arabinose which is the inducer of pHERD20T or pHERD20T*mucA*. **B.** Representative alginate levels (μg/ml) produced by *PA* wild-type (white bar), **Δ***mucA* (light gray bar) and *mucA22* (black bar) bacteria, respectively (n = 3). **C.** Time course of aerobic mucoid reversion profile of *mucA22* (solid line) vs. **Δ***mucA* (dashed line) bacteria (n = 3).

Isogenic *mucA1* and **Δ***mucA* mutants in the PAO1 background were visibly mucoid on L-agar plates (Fig 2A). The **Δ***mucA* mutant (Δ157–194) was created in both the Hassett and Schurr labs and confirmed by sequence analysis, S1A–S1C Fig). However, we must emphasize that a complete in-frame deletion could not be constructed despite numerous attempts by both laboratories and equipped with the appropriate mutagenesis constructs. Additionally, the *PA mucA22* and **Δ***mucA* mutants were complemented *in trans* to the nonmucoid phenotype using the wild-type *mucA* gene inserted into the arabinose-inducible pHERD20T plasmid (Fig 2A). Surprisingly, the **Δ***mucA* mutant was found to generate significantly less alginate than *mucA22* (*p* = 0.012) but greater than the nonmucoid strain, PAO1 (Fig 2B). We also tested the alginate stability phenotype in *mucA22* vs. **Δ***mucA* bacteria for it is well known that the process of mucoid reversion (mucoid-to-nonmucoid phenotype) occurs when bacteria are grown under aerobic static conditions [56]. Fig 2C indicates that mucoid stability is greater in *mucA22* versus **Δ***mucA* bacteria after incubated for 48 hr.

### Mucoidy and A-NO_2_^-^ planktonic sensitivity of *mucA22*, Δ*mucA*, and strain FRD1 (*mucA22*) relative to *PA*

**Fig 3:**
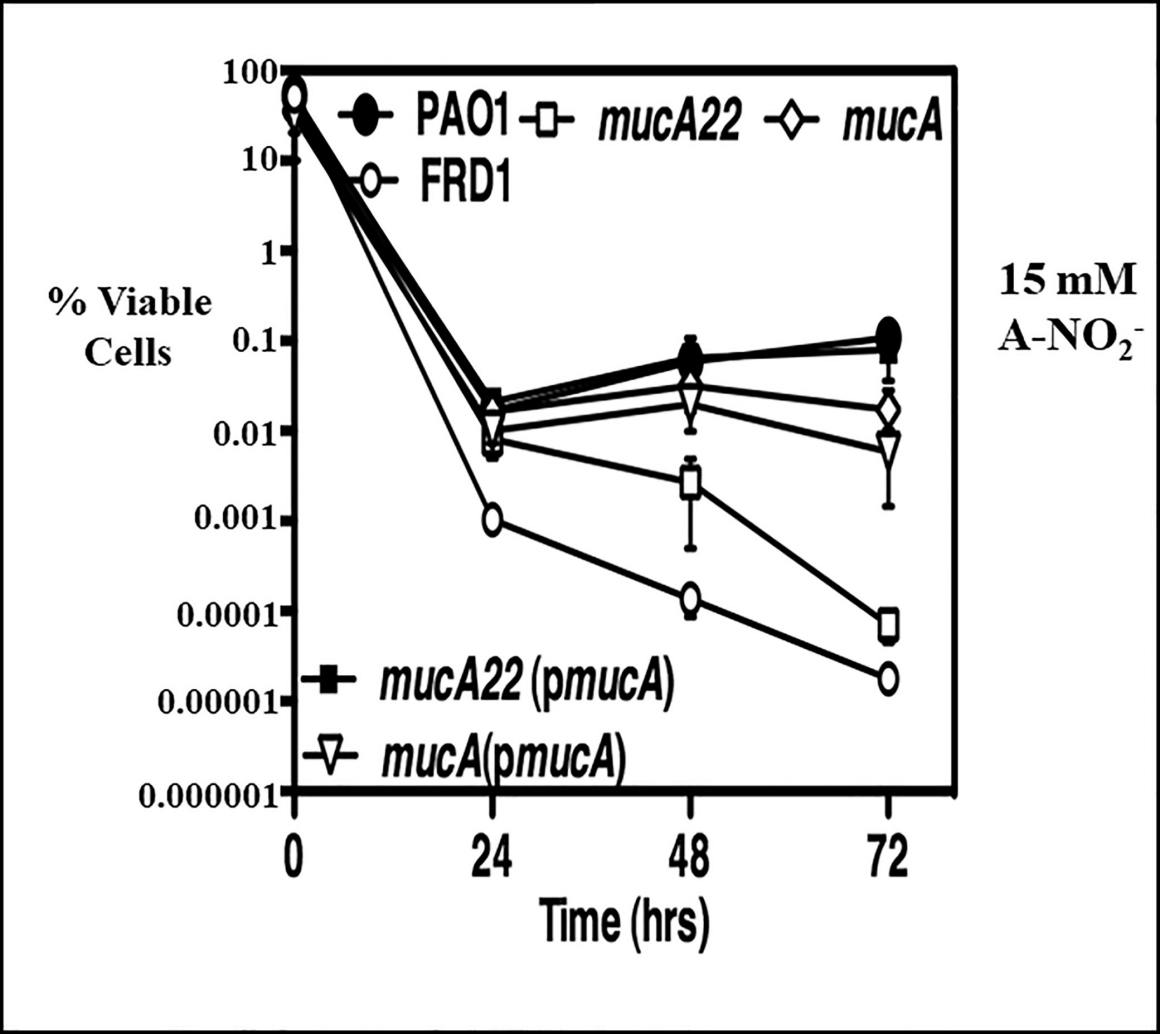
Anaerobic A-NO_2_^-^ susceptibility assay in planktonic bacteria. Overnight planktonic cultures of *PA* wild-type strain PAO1 (filled circles), *mucA22* (pHERD*mucA*) (closed squares), **Δ***mucA* (open diamonds), Δ*mucA* (pHERD*mucA*) (open inverted triangle), *mucA22* (open squares) as well as the A-NO_2_^-^ sensitive CF isolate FRD1 (closed circles) were diluted 1:100 into fresh LBN, pH 6.5 supplemented with 15 mM NaNO_2_, respectively. Cultures were incubated anaerobically and sampled at the indicated times for CFU enumeration (n = 3).

The sensitivity of each strain used in Fig 2A–2C to A-NO_2_^-^ in anaerobic planktonic cultures was assessed, as well as the well-known CF *mucA22* isolate known as FRD1 [9]. Surprisingly, the **Δ***mucA* mutant was found to be more *resistant* to A-NO_2_^-^ relative to a sensitive *mucA22 PA* mutant. Strain FRD1 was even more sensitive to 15 mM A-NO_2_^-^ than its *mucA22 PA* PAO1-based mutant counterpart (Fig 3).

### A-NO_2_^-^ sensitivity of *mucA22* vs. Δ*mucA* in anaerobic biofilms

**Fig 4:**
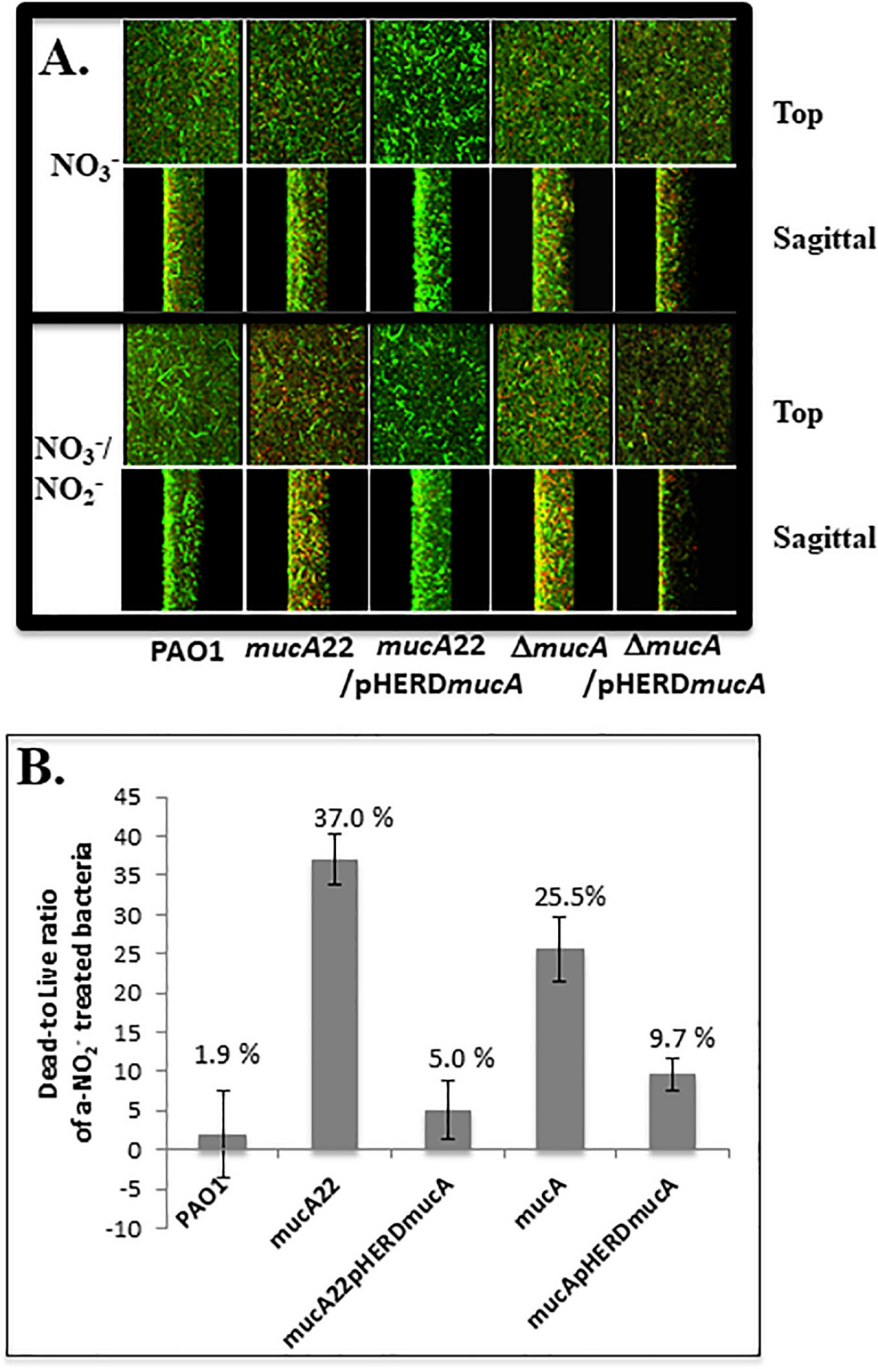
**(A) Assessment of A-NO**_**2**_^**-**^ **on *PA* (wild-type), *mucA22* and Δ*mucA* mutant strains viability when grown anaerobically as biofilms.** Bacterial suspensions were diluted 100-fold in LBN, pH 6.5 and grown in Costar glass-bottomed, chambers amenable to confocal laser scanning microscopic analysis. The planktonic cells were washed from one-day old biofilms. The bacterial biofilms were separated into 2 conditions; control (LBN (NO_3_^-^), pH 6.5) and treated (LBN (NO_3_^-^), pH 6.5 plus 15 mM A-NO_2_^-^ or NO_3_^-^/NO_2_^-^) conditions and continuously grown for 2 more days under anaerobic condition. Both top and sagittal views of each live/dead stained biofilm are depicted after CLSM. Live cells are green while red cells are dead **(B)** The ratio of percent cells dead was calculated by comparison between control and treated conditions. The experiments were performed 3 times independently (n = 3).

We have previously shown that anaerobic conditions favor more robust biofilm formation by *PA* than aerobic conditions and that anaerobic conditions can exist in the thick CF airway surface liquid [8], thereby limiting the overall efficacy of the powerful aminoglycoside, tobramycin and other front-line antibiotics [57]. Thus, we next assessed the overall efficacy of A-NO_2_^-^ against mature, pre-formed anaerobic biofilms in strains *PA*, *mucA22*, **Δ***mucA* and their complemented strains. Fig 4A shows confocal laser scanning microscopy (CLSM) analysis results of 1 day old biofilms of bacteria that were used for the planktonic A-NO_2_^-^ susceptibility assay. These bacterial biofilms were continuously grown in LBN-pH 6.5 (control condition, NO_3_^-^) or LBN-pH 6.5 (treated condition, NO_3_^-^ 15 mM NO_2_^-^) for 2 additional days. As shown in the planktonic susceptibility assay, *mucA22* strains were also more sensitive to A-NO_2_^-^ than the Δ*mucA* strain in bacterial biofilms that showed 37% and 25.5% cell death compared to control, respectively (*p* = 0.029, Fig 4A and 4B). A-NO_2_^-^ resistance complementation was achieved using pHERD*mucA*. In some cases, the biofilm structure often was slightly altered, especially in *mucA*-complemented bacteria which may be a function of multiple copies of cellular MucA. Taken together, our results showed that *mucA22* is more sensitive to A-NO_2_^-^ than **Δ***mucA* in both planktonic and biofilm cultures.

### Sensitivity of *mucA22* but not Δ*mucA* PA to A-NO_2_^-^ during chronic murine lung infection

**Fig 5:**
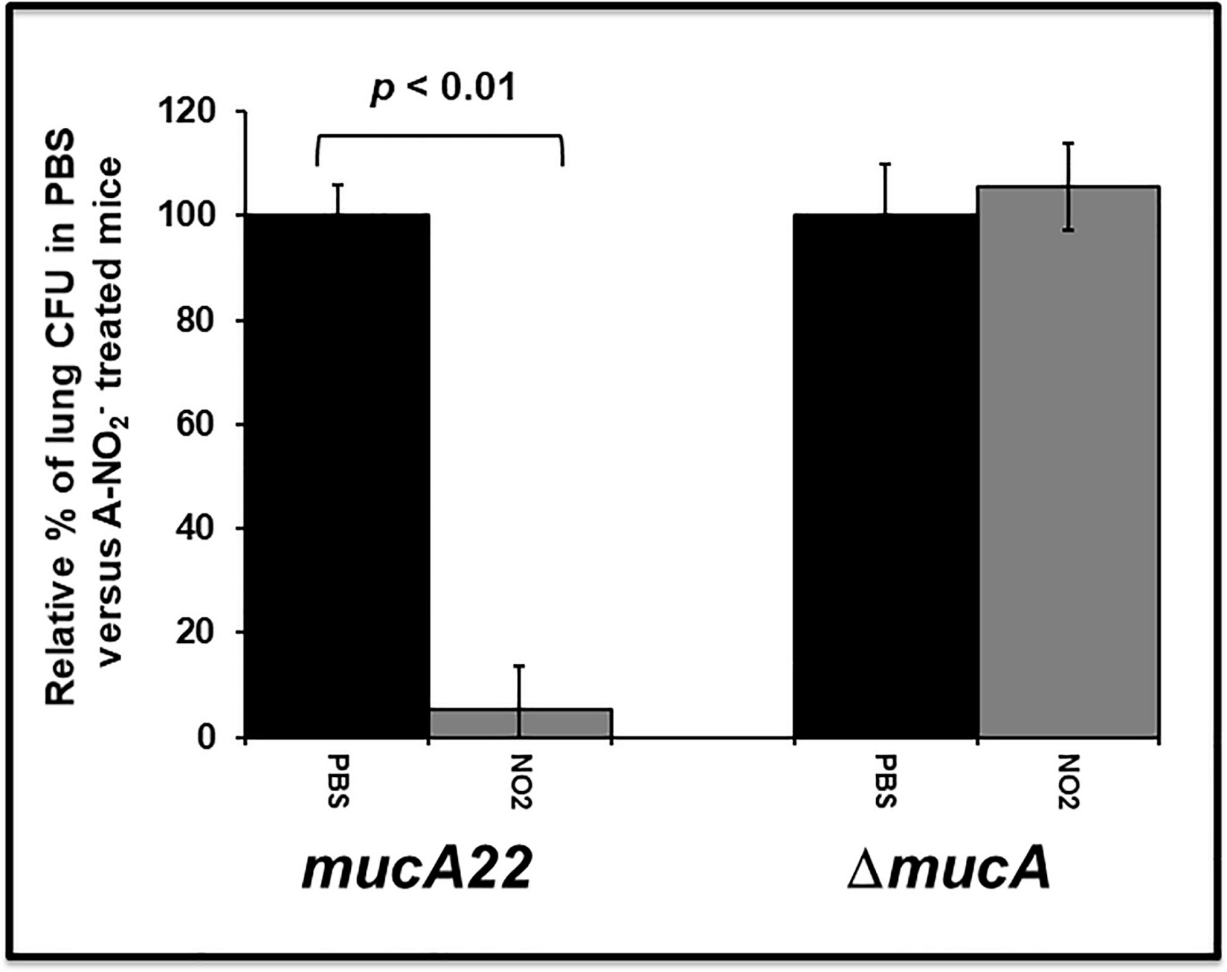
Sensitivity of *mucA22* vs. Δ*mucA PA* during a mouse chronic airway infection. Six-week old male Balb/C mice were infected intratracheally with 50 μl of ~5 x 10^6^ of *mucA22* or **Δ***mucA* mutant strains suspended in 0.9% saline containing purified *PA* alginate (final concentration 1.1 mg/ml). After 24 hr, mouse lungs were instilled with 25 μl of 15 mM A-NO_2_^-^ at pH 6.5 (in 0.1 M phosphate buffer) intranasally twice daily. On day 5 post-infection, the mice were sacrificed, and the viable bacteria from serially diluted lung homogenates were enumerated (n = 8).

To test the role of MucA in A-NO_2_^-^ sensitivity in an animal model of infection, a chronic murine lung infection model was employed as previously described with the exception that strain PAO1 was not used for it is nonmucoid with a wild-type *mucA* allele [9]. Infected mice were treated twice daily with 15 mM A-NO_2_^-^ for 5 days and the mice were then sacrificed. Fig 5 demonstrates that *mucA22* were very sensitive to A-NO_2_^-^ treatment when compared to **Δ***mucA* mutant bacteria. This is consistent with the planktonic and biofilm A-NO_2_^-^ susceptibility assay results.

### Microarray analysis of wild-type, *mucA22* and Δ*mucA PA* upon exposure to A-NO_2_^-^

**Fig 6:**
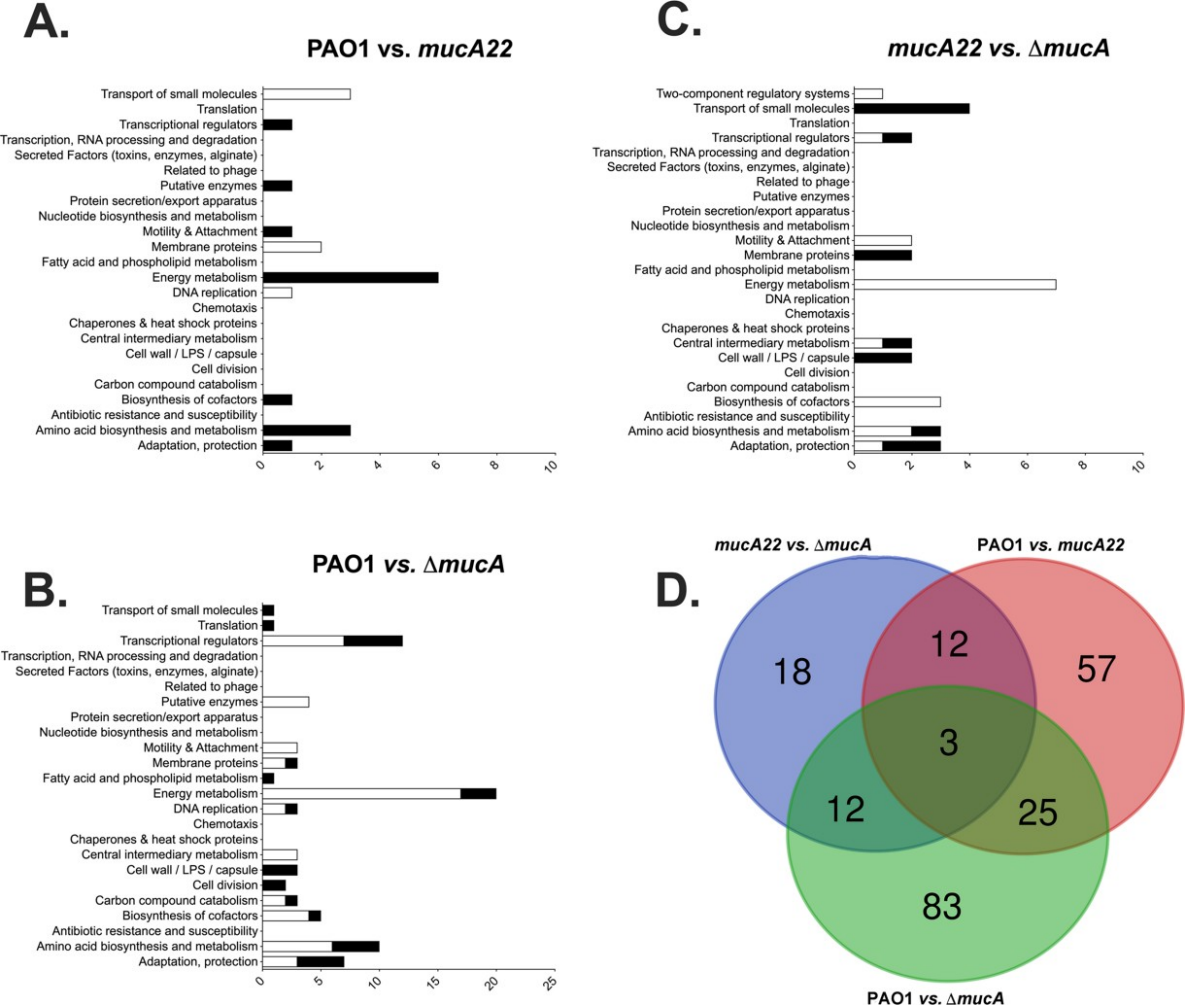
**Microarray assessment of gene classes that are activated (white bars) vs. repressed (black bars) in (A) wild-type PAO1 vs. *mucA22*, (B) PAO1 vs. Δ*mucA* and (C) *mucA22* vs. Δ*mucA* bacteria and (D) Venn diagram indicating gene expression patterns that overlap with A-NO**_**2**_^**-**^**-treated anaerobic PAO1, *mucA22* and Δ*mucA* strains.** The colors and the appropriate genes within each color subset are listed in Table 2, respectively.

Given that *mucA22* mutant bacteria were more sensitive to A-NO_2_^-^ than Δ*mucA* bacteria in planktonic and biofilm culture as well as during murine airway infection, we next performed transcriptional profiling experiments using wild-type PAO1, *mucA22* and Δ*mucA* bacteria to help elucidate the mechanism(s) underlying this apparent paradox. First, considerable efforts were made to isolate quality RNA from A-NO_2_^-^ treated organisms. After many attempts and various protocol adjustments, all three strains were grown for 24 hr under anaerobic conditions in LBN, pH 6.5, followed by treatment of each organism with 15 mM A-NO_2_^-^ for 20 min. The number of genes in each functional class comparing (i) PAO1 vs. *mucA22* (Fig 6A, S1 Table), (ii) PAO1 vs. Δ*mucA* (Fig 6B, S2 Table) and (iii) *mucA22* vs. Δ*mucA* (Fig 6C, S3 Table) are depicted, respectively. Interestingly, genes involved in NIR and NOR activity showed significantly reduced expression in *mucA22* bacteria anaerobic induction conditions, while the expression of these genes was elevated in the Δ*mucA* mutant. This is consistent with the A-NO_2_^-^ sensitivity phenotype of the *mucA22* strain as this could lead to accumulation of toxic NO_2_^-^/NO in the bacteria as a result of inefficient use of the anaerobic respiratory pathway involved in A-NO_2_^-^ metabolism: A-NO_2_^-^ > NO > N_2_O. Fig 6D is a Venn diagram synopsis of gene overlap, the colors of which and the genes are also shown in Table 2.

**Table 2:** Overlapping differential gene expression corresponding to Fig 6D*.

	PA0025_aroE_at	PA0523_norC_at	PA0525_at	PA1059_at	PA1083_flgH_at	PA1837_at
A.(18)	PA1838_cysI_at	PA2687_pfeS_at	PA3413_at	PA3530_at	PA3630_at	PA3632_at
	PA3880_at	PA4155_at	PA4156_at	PA4158_fepG_at	PA4161_fepG-at	PA4896_at
	PA0126_at	PA0291_oprE_at	PA0293_at	PA0320_at	PA0425_mexA_at	PA0431_at
	PA0548_tktA_at	PA0549_at	PA0792_prpD_at	PA0793_at	PA0794_at	PA0795_prpC_at
	PA0796_prpB_at	PA0797_at	PA1135_at	PA1136_at	PA1137_at	PA1239_at
	PA1805_ppiD_at	PA2015_at	PA2287_at	PA2288_r_at	PA2657_at	PA2659_at
	PA2840_at	PA2934_at	PA3008_at	PA3118_leuB_at	PA3119_at	PA3120_leuD_at
B.(57)	PA3195_gapA_at	PA3748_at	PA3787_at	PA3932_at	PA4030_at	PA4059_at
	PA4129_at	PA4130_at	PA4131_at	PA4132_at	PA4234_uvrA_at	PA4636_at
	PA4768_smpB_at	PA4769_at	PA4839_speA_at	PA4853_fis_at	PA4915_at	PA4932_rplI_at
	PA4933_at	PA4935_rpsF_at	PA5125_ntrC_at	PA5147_mut_at	PA5162_rmlD_at	PA5136_rmlA_at
	PA5304_dadA_at	PA5383_at	Ig_326671_3272284_at			
	PA0069_at	PA0082_at	PA0083_at	PA0124_at	PA0161_at	PA0224_at
	PA0286_at	PA0353_ilvD_at	PA0410_pilI_at	PA0412_pilK_at	PA0413_at	PA0526_at
	PA0558_at	PA_0669_at	PA0670_at	PA0731_at	PA0765_mucC_at	PA0906_at
	PA0910_at	PA0911_at	PA0933_ygcA_at	PA0938_at	PA1048_at	PA1431_rsaL_at
	PA1541_at	PA1588_sucC_at	PA1589_sucD_at	PA1746_at	PA2277_arsR_at	PA2290_ged_at
	PA238-1_at	PA2445_gcvP2_at	PA2639_nuoD_at	PA2640_nuoE_at	PA2641_nuoF_at	PA2642_nuoG_at
	PA2643_nuoH_at	PA2646_nuoK_at	PA2648_nuoM_at	PA2664_fhp_at	PA2667_at	PA2705_at
C.(83)	PA2706_at	PA2718_at	PA2788_at	PA2796_tal_at	PA2846_at	PA2869_at
	PA2946_at	PA2948_cobM_at	PA3011_atopA_t	PA3012_at	PA3013_foaB_at	PA3066_at
	PA3067_at	PA3181_at	PA3392_nosZ_at	PA3471_at	PA3567_at	PA3575_at
	PA3620_mutS_at	PA3859_at	PA3972_at	PA3973_at	PA4006_at	PA4007_proA_at
	PA4045_at	PA4046_at	PA4061_at	PA4068_at	PA4180_at	PA4440_at
	PA4493_at	PA4499_at	PA4625_at	PA4803_at	PA4812_fdnG_at	PA4919_pncB1_at
	PA5479_gltP_at	PA5496_at	PA5557_atpH_at	PA5564_gidB_at	PA5565_gidA_at	
	PA0510_at	PA0515_at	PA0561_nirF_at	PA0518_nurM_at	PA0520_nirQ_at	PA0764_mucB_at
D.(12)	PA0766_mucA_at	PA1423_lasI_at	PA2830_htpX_at	PA3971_at	PA4810_fdnI_at	PA5429_aspA_at
	PA0201_at	PA0280_cysA_at	PA0281_cysW_at	PA0283_sbp_at	PA0284_at	PA0396_pilU_at
E.(12)	PA0524_norB_at	PA2599_at	PA3446_at	PA3450_at	PA3931_at	PA4443_cysD_at
	PA0045_at	PA0179_at	PA0432_sahH_at	PA0546_metK_at	PA0547_at	PA0671_at
	PA1132_at	PA1423_at	PA1587_lpdG_at	PA1865_at	PA2644_nuoI_at	PA2658_at
F.(25)	PA2662_at	PA2663_at	PA3472_at	PA3551_algA_at	PA3747_at	PA4033_at
	PA4630_at	PA4971_at	PA5203_gshA_at	PA5250_at	PA5251_at	PA5252_at
	PA5483_algB_at					
G.(3)	PA0517_nirC_at	PA0519_nirS_at	PA0807_at			

*Overlapping gene groups (probe set ID) are colored as the Venn diagram showing in Fig 6D.

### Identification of *S*-nitrosylated proteins in A-NO_2_^-^-treated bacteria

To further evaluate the response of *PA* to A-NO_2_^-^, a unique proteomic approach was used to measure cysteine *S*-nitrosylation of cellular proteins mediated by NO generated from A-NO_2_^-^reduction, known as SNOSID (***S***-**n**itr**o**sylation, **SNO S**ite **Id**entification, [58]). A-NO_2_^-^ treatment of bacteria allows for significant generation of what are termed ***S***-**n**itr**o**sylated proteins (or SNO proteins, [59]). This technique was used to monitor *PA* proteins that had increased or decreased levels of SNO-proteins in wild-type, *mucA22* and Δ*mucA* strains. The data collected are based upon SNO proteins in the 2-D gels shown in S2A–S2C Fig. Twelve SNO proteins are presented in tabular form after mass spectrometric identification and their relative levels expressed in each of the aforementioned strains listed in Table 3. Interestingly, several of the proteins that showed differing levels of *S*-nitrosylation include those involved in anaerobic metabolism (NirQ, NirS, NrdJB) or anaerobic survival (UspK, FdnG), alginate production (GDP-mannose dehydrogenase), the TCA cycle (succinate dehydrogenase, SdhB) and virulence (Vfr).

**Table 3:** Identification of SNO proteins from the different bacteria used in this study. For example, the fold change in spot 2A (FdnG) in the *mucA*22 mutant is 2.2-fold more *S*-nitrosylated than wild-type bacteria.

Spot ID	Gene/protein name			Fold	change*		
		*mucA22* vs	PAO1	Δ*mucA* vs	PAO1	Δ*mucA* vs	*mucA22*
		Up	Down	Up	Down	Up	Down
2A	*fdnG*	2.2	-	2.4	-	-	-
2B	*fdnG*	13	-	6.3	-	-	2.1
5	*ygbM*	-	-	-	-	-	1.5
6A	*nrdJB*	-	1.4	-	-	1.3	-
6B	*nrdJB*	-	1.9	-	1.6	1.2	-
7	*priC* or *opdA*	1.7	-	1.7	-	-	-
8	*algD*	-	-	1.5	-	1.5	-
10	*uspK*	-	1.7	-	2.4	-	1.4
11	*Vfr*	-	6.7	-	7.1	-	
13	*nirS*	-	-	-	2.3	-	2.1
15	*oprH*	-	3.2	-	-	2.9	-
17	*prpB* or *bcpA*	-	1.2	1.4	-	1.7	-
18	*nirQ*	1.7	-	-	-	-	1.7
19	*galU*	-	1.4	-	1.3	-	-

* = Relative SNO protein expression levels.

**Fig 7:**
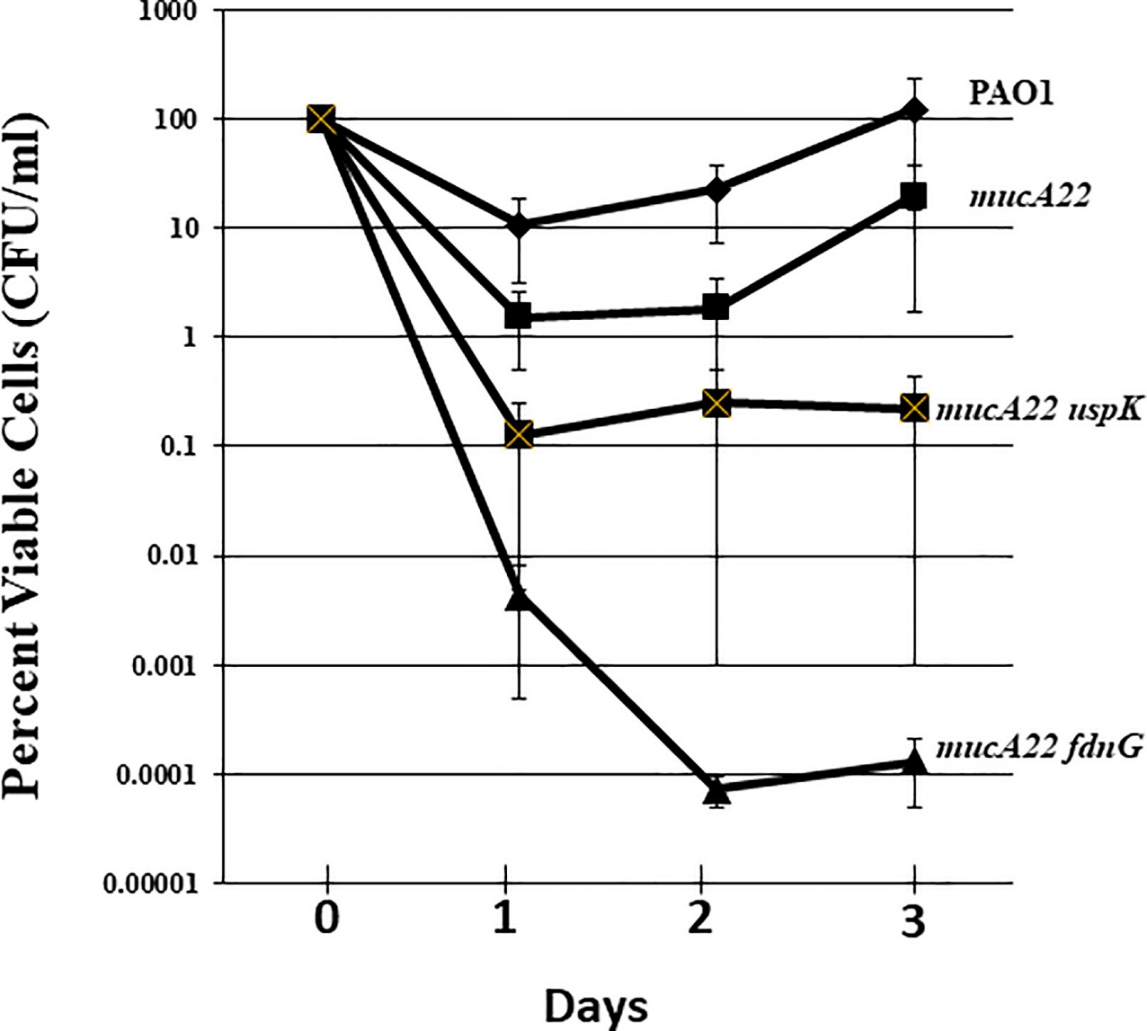
A-NO_2_^-^ sensitivity of anaerobic planktonic PAO1 (diamonds) *mucA22* mutant (squares) with secondary mutations in *uspK* (strikethrough squares) and *fdnG* (triangles). We must note that there were no observable differences in the growth patterns of *mucA22* and the double mutants.

Given that *PA usp* (**u**niversal **s**tress **p**rotein) genes are necessary for long-term anaerobic survival [60], we postulated that a double *mucA22 uspK* would be hyper-sensitive to anaerobic A-NO_2_^-^ treatment. Fig 7 clearly confirms our hypothesis that this mutant is indeed more susceptible to A-NO_2_^-^ treatment than *mucA22* mutant bacteria. We also identified that the FdnG (formate dehydrogenase-O, major subunit, [61]) that is physically associated with NAR *in vivo* [62], was identified to be *S*-nitrosylated. In comparison to the *mucA22 uspK* mutant, the *mucA22 fdnG* double mutant was even more sensitive to A-NO_2_^-^ (Fig 7).

### Why is CF clinical strain FRD1 hypersensitive to A-NO_2_^-^ relative to PAO1 *mucA22*?

**Fig 8:**
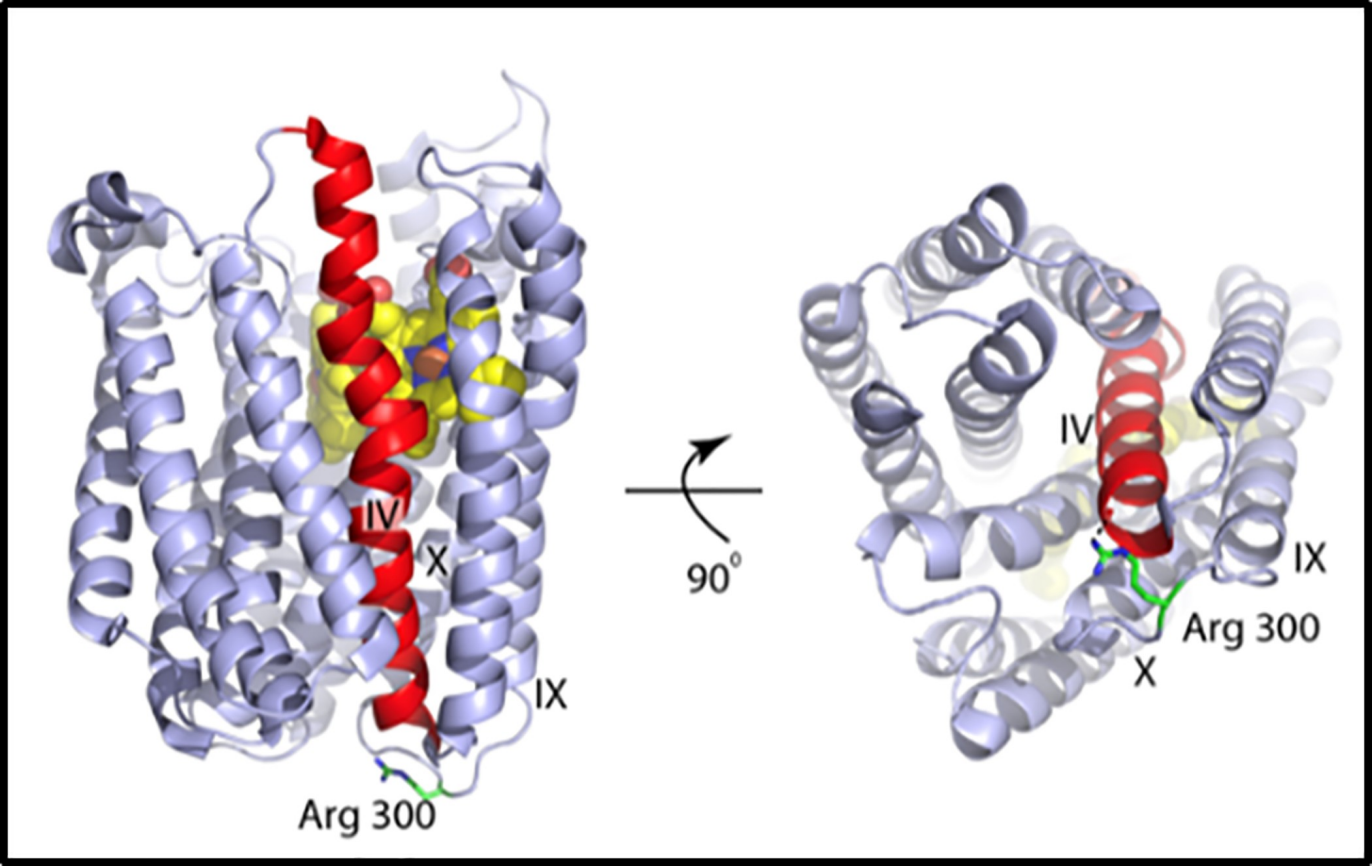
Position of Arg300 in the structure of PA NorB. Ribbon representation of NorB (PDB:3o0r) [65] in two orientations. Arg300 is show in stick format. Helix IV is colored red and the heme centers are show in spacefill. Arg300 hydrogen bonds to the mainchain carbonyl of Lys288 is shown in the right panel.

To determine whether mucoid CF strain FRD1 (i.e., a well-studied, chronically adapted CF strain, [51]) had mutations other than *mucA22* that might reveal clues as to why it is so sensitive to A-NO_2_^-^ relative to *PA mucA22*, we assessed differences in the *PA* vs. FRD1 genome, the latter of which was recently sequenced [63]. Specifically, we assessed potential mutations in genes encoding anaerobic respiratory/regulatory genes in strain FRD1 that are not in strain *PA* [63]. The PAO1 genome is comprised of 6,264,404 bp while that of the FRD1 genome has 6,712,339 bp total nucleotides, with a percent G+C content of ~66%, based upon data compiled from 133 contigs [63, 64]. A search and analysis of genes predicted to be involved in A-NO_2_^-^, resistance showed numerous single base pair substitutions. These included missense mutations in genes such as several of the *nar* genes, as well as in *nirS*, and *norCB* among others that would hypothetically affect A-NO_2_^-^, sensitivity. However, during anaerobic conditions, mutations within the *norB* or *norC* genes would likely influence sensitivity to A-NO_2_^-^. Differences in the coding sequence of the PAO1 and FRD1 *norB* gene revealed a deletion of Arg300 in the latter [63]. In strain PAO1, Arg300 is positioned in a highly basic loop that consists of 4 consecutive arginine residues linking Helix IX and Helix X on the cytoplasmic side (Fig 8). Arg300 forms a hydrogen bond with the carbonyl of Lys228 that caps the C-terminus of Helix VI. Therefore, it is possible that loss of this interaction might result in conformational changes that distort the binuclear centers of NorB, specifically His207, which is positioned on Helix VI and is a ligand for Fe_B_. When cloned into a PAO1 *norCB* mutant, the FRD1 *norCB* genes could not rescue a normal anaerobic growth density (S4 Table).

## Discussion

### Anaerobic A-NO_2_^-^ sensitivity: intuitive and nonintuitive gene products involved in its defense

The *mucA22* allele in *PA* is the most common mutation (deletion of a C residue at position 430) found during the course of chronic CF and COPD, resulting in a truncated MucA protein of ~15.8 kDa and resulting mucoid conversion (alginate-overproduction). A little over 13 years ago, we discovered that mucoid, *mucA22* mutant *PA* were uniquely susceptible to A-NO_2_^-^ at the slightly acidic pH that is consistent with that of the CF (pH 6.4–6.5 [9, 66] and COPD airway surface liquid [67], effectively revealing a previously unrecognized “Achilles heel” of these highly refractory organisms. This weakness was a significant translational development given that *mucA22* organisms are formidably antibiotic- and phagocyte-resistant. Prior to that work, two seminal papers describing the anaerobic biofilm mode of growth in CF were published in 2002 [8, 13]. Since then, dedicated research efforts have ensued to unravel the biology of the ever-evolving organisms cultured from anaerobic CF and COPD airway biofilms. In addition, the slightly acidic nature of CF and COPD airways allows for A-NO_2_^-^ to be reduced to NO, that we have shown is known to kill not only *mucA22* mutant *PA*, but also *S*. *aureus* (MRSA) and *B*. *cepacia* [29].

Thus, given these clinically relevant and translational developments (U.S. Patent # 8,557,300 B2 by corresponding author), the primary goal of this study was to identify additional genes involved in A-NO_2_^-^ sensitivity or resistance. Using both transposon and strategic insertion or deletion mutagenesis approaches, a series of mutants displaying the aforementioned traits were discovered. First, we showed that the major catalase, KatA, is required for anaerobic A-NO_2_^-^ resistance because of its surprisingly inherent ability to buffer the NO derived from A-NO_2_^-^ reduction [48]. Second, a mutant defective in the *rhl* quorum sensing circuit regulator (*rhlR*), that we have also found previously to commit an anaerobic suicide by overproduction of endogenous respiratory NO in biofilms [8], was also found to be sensitive to exogenous anaerobic A-NO_2_^-^. Other genes that were necessary for optimal A-NO_2_^-^ resistance included another quorum sensing regulator gene, *pmpR* (regulator of the ***P****seudomonas* **q**uinolone **s**ignal (PQS), [68]), *lon* (encoding the Lon protease), PA4455 (a putative ABC transporter, [47]), and *nrdJa*,*b* (encoding anaerobic ribonucleotide reductase). The Lon protease has previously been shown to be required for optimal activity of Fhp (flavohemoglobin), an aerobically expressed flavohemoglobin with aerobic but not anaerobic NO detoxifying properties [69, 70]. Thus, it makes perfect sense that the *PA0779* (*asrA*, a Lon peptidase) mutant is also sensitive to A-NO_2_^-^. However, the most intriguing results of this study and given the predominance of *mucA* mutations from predominantly CF and to a lesser extent COPD were from A-NO_2_^-^ sensitivity and insensitivity results comparing *mucA22* vs. Δ*mucA* bacteria, the latter of which was found to be paradoxically ***resistant*** to it in planktonic, biofilm and during a chronic murine lung infection. Thus, the remainder of this study focused on understanding the mechanism underlying this puzzling development.

### Microarray and SNO protein analyses and interpretations upon exposure of bacteria to A-NO_2_^-^

To better understand why wild-type and Δ*mucA* strains were less sensitive to A-NO_2_^-^ than *mucA22* bacteria, we strategically performed transcriptional profiling of wild-type, *mucA22* and Δ*mucA* strains exposed to anaerobic A-NO_2_^-^. We found that wild-type bacteria and the Δ*mucA* mutant had far higher transcriptional levels of protective *nir* (encoding NIR), *nor* (encoding NOR) and *fhp* (encoding flavohemoglobin) genes when compared to the very low transcription of such genes in the *mucA22* mutant. This is consistent with the very low transcription of *nir* and *nor* genes in anaerobic clinical isolate FRD1 (a *mucA22* mutant) exposed to A-NO_2_^-^ as we have described previously [9]. In addition, many *nuo* genes (encoding NADH dehydrogenase) were also upregulated in the Δ*mucA* mutant. It is not surprising that the *nuoABDFHIJKMN* genes are also required for anaerobic growth with both NO_3_^-^ and NO_2_^-^ as terminal electron acceptors [71, 72]. Thus, the wild-type and Δ*mucA* mutant have a greater ability to detoxify both NO_2_^-^ and NO. Another interesting observation from the transcriptional profiling was that wild-type PAO1 and the *muc22* mutant have normal expression of *mucD*, the last gene of the *algT/U* operon, but the Δ*mucA* mutant has 5-fold less expression. MucD is a periplasmic protease that serves as a weak negative regulator of AlgW protease, which senses periplasmic stress, leading to cleavage/activation of MucA [73]. This alteration in the AlgT(U) (σ^22^) pathway might affect expression of *nir*, *nor*, and *fhp* genes, which is currently under investigation. However, the Δ*mucA* strain has 27 extra amino acids than the *mucA22* mutant, begging the question as to whether these amino acids are involved in the anaerobic respiratory pathway vis a vis protection against NO.

To complement our transcriptomic data, our *S*-nitrosylation analysis also revealed mechanistic clues from the protein level. As a reminder, *S*-nitrosylation is a mechanism of signal transduction in eukaryotes based upon a covalent bond between NO and cysteine residues, thereby serving as a post-translational modification [74]. In bacteria, however, *S*-nitrosylation can actually be used to trigger anaerobic gene transcription mediated, as seen by SNO-mediated activation of OxyR in *E*. *coli* [75]. The most *S*-nitrosylated proteins (an event that can compromise protein function) have significant links to the overall anaerobic respiratory cascade of *PA*. These include NrdG (a regulator of *nrdJa*,*b* genes), NirS (NIR), NirQ [76], NrdJa (anaerobic ribonucleotide reductase (RNR)) and NuoL (NADH dehydrogenase chain L). Other than *nirS*, it should be noted that all of the aforementioned genes are essential for anaerobic growth [77], especially the Class II RNRs [78]. Thus, we believe that S-nitrosylation is a consequence of exposure to A-NO_2_^-^. However, we also believe that the NO generated by A-NO_2_^-^ reduction overwhelms the machinery involved in the transcription of the protective *norCB* genes (encoding NO reductase). In 2007, we showed that high (~16 μM) endogenous levels of NO inactivate the ANR/DNR regulatory cascade in a *norCB* mutant, leading to abysmally poor growth under anaerobic conditions [50].

### What could be the mechanism of A-NO_2_^-^ toxicity in anaerobic *mucA22* mutant PA relative to that of a Δ*mucA* mutant?

There are at least five possible mechanisms of cell injury and death from A-NO_2_^-^ in *mucA22* mutant bacteria, (a) inhibition of heme-enzyme(s), (b) destruction of iron-sulfur (Fe-S) centers (e.g., the master anaerobic transcriptional regulator ANR, an NO-sensitive 4Fe-4S cluster protein, [50]), (c) disruption of cellular iron homeostasis (with a,b,c being primarily via the formation of dinitrosyliron complexes [48]), (d) oxidative injury (upon introduction to an aerobic environment), and (e) protein adduction (nitration, nitrosation). NO reacts with only two groups of species under biological conditions, other radicals (such as O_2_ and O_2_^•-^) and transition metals. At first glance (however *vide infra*), oxygen radical-based death can be ruled out because of the strict anaerobiosis enforced in these studies given the fact that both CF and COPD airways have anaerobic airway pockets, leaving likely transition metal interactions as the major mechanism(s) of NO toxicity. Most cellular metal ions are “shielded” from NO and thus are not likely targets. The “classic” metal targets are iron and heme-containing (those with an open ligation position), Fe-S centers, and the “chelatable iron pool” (CIP) [79–84].

### Final questions that urgently need answers

**Fig 9:**
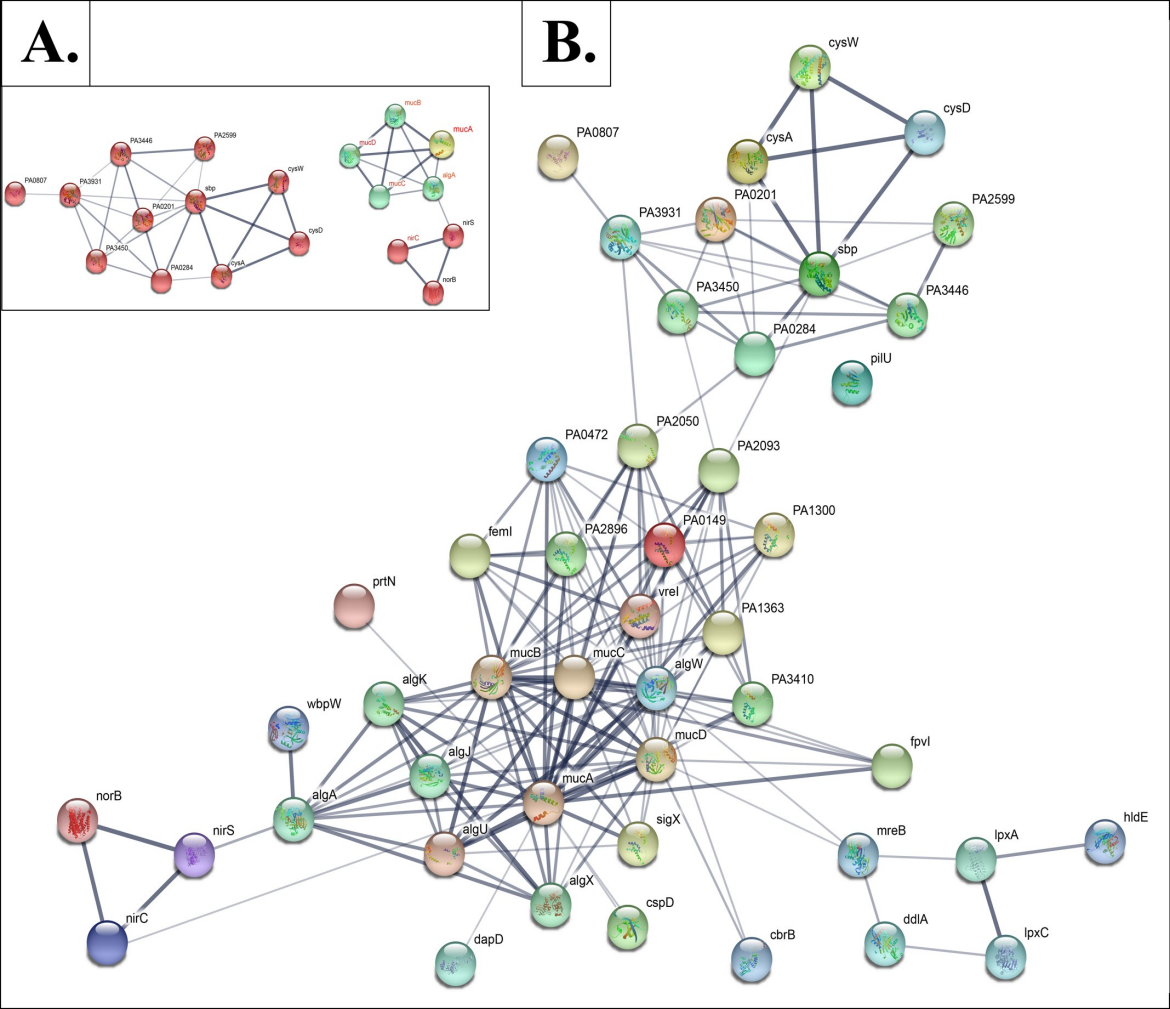
Integrated protein interaction networks. **A. Inset. Simplified version of the integrated protein interaction networks. B.** Fifteen proteins encoded by the genes involved in A-NO_2_^-^ sensitivity (green nodes; overlapping DEGs of *PA* vs *mucA22* and Δ*mucA* vs *mucA22*) and 32 predicted MucA interactors (red nodes) were used to build an integrated protein interaction network. The genes labeled in red were also found differentially expressed between *PA* vs Δ*mucA*.

**What might be the biological functions of MucA vs. MucA22: are there potentially other binding partners during anaerobic growth other than the extracytoplasmic sigma factor, AlgT(U) and the periplasmic negative regulator MucB?** The most intriguing finding of this work was the discovery that *mucA22* and Δ*mucA* bacteria behave very differently *vis a vis* A-NO_2_^-^ susceptibility patterns. Our results suggest that the 15.8 kDa truncated MucA22 protein has an unknown function that is possibly to dysregulate the anaerobic respiratory regulatory and enzymatic genetic circuitry. In support of this notion, and based upon this pioneering work, we have previously shown that NIR and NOR genes are dramatically down-regulated in *mucA22* bacteria relative to wild-type organisms [9]. As a final attempt to build the hypothesis-driven platform for a future study, we elected to use bioinformatic techniques to elucidate further clues as to why mucoid *mucA* mutants are more sensitive to A-NO_2_^-^ than wild-type, nonmucoid bacteria. To accomplish this goal, our microarray data were analyzed to identify differentially expressed genes (DEGs) between three conditions (anaerobic *PA*, *mucA22* and Δ*mucA* exposed to 15 mM A-NO_2_^-^, pH 6.5 in pairs. The significant DEGs were selected by the criteria that fold change values are larger than two and the *p*-value is lower than 0.05. We also compared the significant DEGs under each comparison design based upon the microarray data and looked for overlapping genes to investigate if the transcriptional profile changes were contributing to the hypersensitivity of A-NO_2_^-^ in *mucA22* mutant bacteria when compared to wild-type and Δ*mucA*). The functional annotation clustering of the gene set was conducted by DAVID to see if any functional pathway was related with alternative gene expression. If the overlapping genes were also found in the *mucA22* vs. Δ*mucA* analyses, it indicates that the genes expressed differently should have a tight interaction with MucA. To elucidate the mechanism underlying A-NO_2_^-^ sensitivity as well as identifying potential interacting partners of MucA, we searched an on-line *PA* PAO1 protein-protein interaction (PPI) database (http://research.cchmc.org/PPIdatabase) using the query word MucA. This database contains prediction results by a random forest classifier that was trained on nine genomic features (co-essentiality, co-expression, co-functionality, co-localization, domain-domain interaction, co-pathway involvement, transmembrane helices, co-operon and co-gene cluster involvement, [85]). This assessment resulted is a large-scale PPI network in *PA* with significant coverage and high accuracy, i.e., 57,746 potential protein interactions covering 4,256 *PA* PAO1 proteins [86]. Among the DEGs, we selected the potential PPI partners of MucA from the interactome database. By querying MucA in the database, we found 17 proteins with which it is predicted to interact (S5 Table, Fig 9). We also searched the STRING database (https://string-db.org) for MucA interactors. The top 20 interactors of high confidence interaction with MucA were used for the following analysis. Five proteins are in common between the interactors predicted by two sources. In total, 32 MucA interactors were predicted (S6 and S7 Tables). We then investigated if these interactors changed expression in the Δ*mucA* or *mucA22* mutants compared to wild type PAO1. Five interactors were found significantly altered expression in *PA* vs Δ*mucA*, including three *muc* genes (*mucBCD*), *algA* and *nirC*, respectively. In contrast, when PAO1 gene expression was compared to the *mucA22* mutant, only levels of *algA* and *nirC* transcription were found significantly changed. Interestingly, only the *nirC* gene was found differentially expressed in all three strains comparisons. In fact, *nirC* expression was highest in the *mucA22* mutant and down-regulated in the Δ*mucA* mutant when compared to PAO1 expression. This pattern indicates that the expression of the *nirC* gene is more likely to be influenced by any changes in the *mucA* gene, and the expression level may be correlated to the A-NO_2_^-^ sensitivity phenotype. We identified possible anaerobic binding partners for MucA, NirC and NirM. Both NirC and NirM are periplasmic *c*-type cytochromes that are known to donate electrons to NIR, thereby promoting efficient anaerobic respiration [87]. These data indicate that there is a distinct possibility that anaerobic MucA22 has a function other than binding to AlgT(U) and MucB. Relatedly, an anaerobic protein “interactome” has been shown linking the primary motility protein, flagellin (FliC) with NirS and DnaK [88]. Very complex yet interesting biology was revealed in the aforementioned work. First, a *nirS* mutant could not form a flagellum and as such was impaired in swimming motility. Conversely, if the flagellum and anaerobic respiration are coordinately regulated in an as yet unappreciated pathway, then a *fliC* mutant should be impaired in anaerobic respiration—which was not the case. The other interesting feature between flagellar expression and mucoidy is that *fliC* is repressed by the AlgT(U)-dependent regulator AmzR that directly represses the *fleQ* gene, the product of which is required for *fliC* transcription [89]. We have previously shown that mucoid *mucA22* mutant bacteria grow poorly anaerobically because they harbor dramatically lower respiratory NAR and NIR activity [9].

### Closing remarks

**Fig 10:**
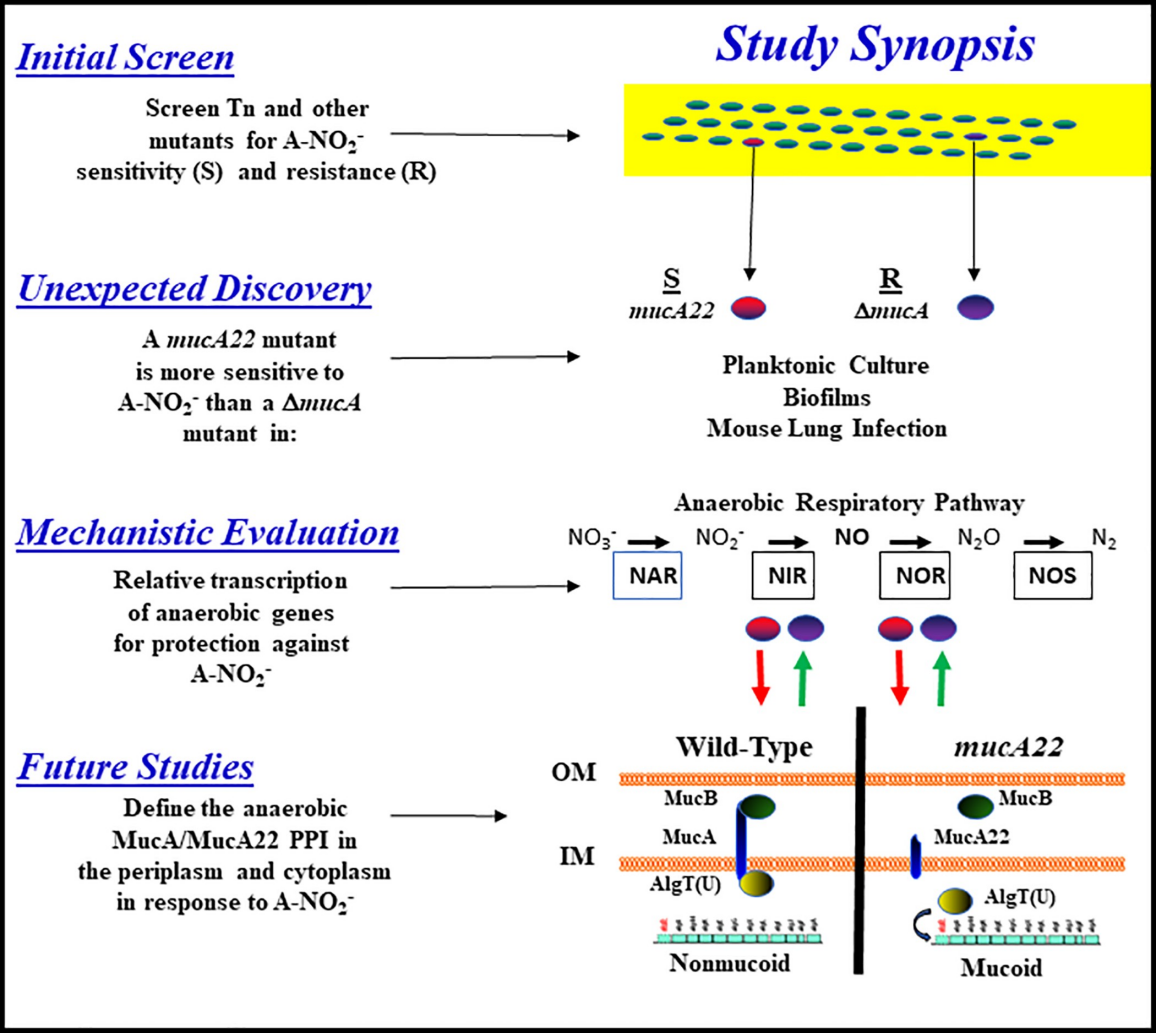
Flow chart of the scientific progress in this study split into (i) initial screen, (ii) unexpected discovery, (iii) mechanistic evaluation and (iv) future studies sections. Green arrows indicate upregulated genes while the red arrows indicate down-regulated genes. NAR, **n**itr**a**te **r**eductase; NIR, **ni**trite **r**eductase; NOR, **NO r**eductase; NOS, **n**itrou**s o**xide **r**eductase. The future studies diagram is an inner (IM) and outer (OM) of nonmucoid and mucoid *PA* with AlgT(U), MucA, MucA22 and MucB in their known cellular locations [90].

Finally, this multi-disciplinary study revealed a fascinating paradox in that Δ*mucA* mutant bacteria possess an anaerobic A-NO_2_^—^resistant phenotype relative to that of *mucA22* mutant bacteria. This is consistent with the myriad of strains (>300) that have been sequenced in several studies indicating that that no mucoid strains would be classified as true deletion mutants and we could not engineer a complete deletion despite numerous attempts [9, 35]. Given the numerous discoveries and scientific disciplines used in this study, we elected to provide a synopsis flow chart that is detailed in Fig 10 as a refresher and eliminate any potential confusion. This figure is divided into (i) initial screen, (ii) unexpected discovery, (iii) mechanistic evaluation and (iv) future studies. Lastly, future studies are designed to identify putative anaerobic MucA and potentially MucA22 binding partners in the context of better understanding the important translational implications of A-NO_2_^-^ treatment for killing of highly refractory airway infectious bacteria in CF and COPD.

## Materials and methods

### Bacterial strains and growth conditions

All bacteria, including newly constructed mutants and plasmids used in this study are listed in Table 4. Organisms were routinely grown in either Luria-Bertani broth (L-broth) or L-broth plus 100 mM KNO_3_ (LBN) or LBN pH 6.5 which is LBN containing 50 mM potassium phosphate. Aerobic cultures were grown at 37°C with shaking at 250 rpm at a 1/10 volume to total Erlenmeyer flask ratio. Media were solidified with 1.5% Bacto-agar. Bacteria were grown under anaerobic conditions at 37°C in a dual-port Coy Laboratories anaerobic chamber. Frozen bacterial stocks were stored at -80°C in a 1:1 mixture of 30% glycerol and stationary-phase bacterial suspension.

**Table 4:** Bacterial strains, plasmids and oligonucleotides used in this study.

Strain, plasmid or oligonucleotide	Description (relevant genotype or phenotype) or sequence (5′ to 3′)	Company source or reference
***E*. *coli***		
DH5α	F^-^ Φ80d*lacZΔM15 endA1 recA1 hsdR17(r*_*K*_^*-*^ *m*_*K*_^*-*^*) supE44 thi-1 gyrA96* Δ*(lacZYA-argF)*U169	Invitrogen
S17-1 λ *pir*	Pro− Res− Mod+ *recA*; integrated RP4-Tet : : Mu-Kan : : Tn*7*, Mob+	[91]
***P*. *aeruginosa***		
PAO1	Wild-type laboratory strain	[92]
FRD1	Mucoid CF clinical isolate with mucA22 allele	[93]
*mucA22*	PAO1 *mucA22* mutant, mucoid	[94]
Δ*mucA*	PAO1Δ*mucA* mutant (Δ157–194) (Hassett and Schurr labs)	This study
*mucA22 uspK*	*mucA22*, *uspK*::*Tn*Gm	This study
*mucA22 fdnG*	*mucA22*, *fdnG*::*Tn*Gm	This study
*moaA2*	*moaA2*::*Tn*Gm	This study
*PA4455*	*PA4455*::Gm	[47]
*PA0964*	*PA0964*::*Tn*Gm	This study
*PA0450*	*PA0450*::*Tn*Gm	This study
*rhlR*	*rhlR*::Gm	[52]
*norCB*	*norCB*::Gm	[50]
*lon*	*lon*::*Tn*Gm	This study
*nuoK*	*nuoK*::*Tn*Gm	This study
**Plasmids**		
pBT20	Mini-Tn delivery vector, Ap^R^, Gm^R^	[95]
pUCGM	Source for Gm^r^ cassette, Ap^R^, Gm^R^	[96]
pEX100T-KS	*Pseudomonas* gene replacement suicide vector with modified multiple cloning site, *sacB*, *oriT*, Cb^R^	[97]
pEXΩ100T	Gm^R^ cassette from pUCGM was inserted into unique *Sca*I site of pEX100T-KS, *sacB*, *oriT*, Gm^R^	This study
pEXΩ100TΔ*mucA*	1kb upstream and downstream fragments flanking *mucA* gene were cloned into pEXΩ100T, *sacB*, *oriT*, Gm^R^	This study
pHERD20T	*Escherichia*-*Pseudomonas* shuttle vector, Ap^R^	[98]
pHERD*mucA*	*mucA* cloned into pHERD20T	This study
**Nucleotides**		
AD2	5′-cangctwsgtntscaa	
Gm447	5′-gtgcaagcagattacggtgacgat	
Gm464	5′-tgacgatcccgcagtggctctc	

Ap^R^, ampicillin resistant; Cb^R^, carbenicillin resistant; Gm^R^, gentamicin resistant

### Manipulation of recombinant DNA and genetic techniques

All plasmid and chromosomal nucleic acid manipulations were performed by standard techniques [99]. Plasmid DNA was transformed into *E*. *coli* strain DH5α (Protein Express, Cincinnati, OH). To detect the presence of insert DNA, X-Gal (5-bromo-4-chloro-3-indolyl-β-D-galactopyranoside; 40 μg/ml) was added to agar media. Restriction endonucleases, the Klenow fragment of DNA polymerase I, T4 DNA polymerase, and T4 DNA ligase were used as specified by the vendor (Invitrogen/ Gibco-BRL Corp., Gaithersburg, MD). Plasmid DNA was isolated by using plasmid miniprep isolation kits (Qiagen), and restriction fragments were recovered from agarose gels by using SeaPlaque low-melting-point agarose (FMC BioProducts, Rockland, ME). PCRs were performed by using Pfu DNA polymerase (BRL) and appropriate primers in an MJ Research thermal cycler, with 30 cycles of denaturation (2 min, 94°C), annealing (1 min, 54°C), and extension (1 min 30 s, 72°C). Amplified DNA fragments were gel purified, cloned into pCR2.1 (Invitrogen), and sequenced.

### Methods used to construct *PA* mutant strains

#### Screening of more sensitive or resistance A-NO_2_^-^ strains using Transposon (Tn) mutagenesis

*PA* strain PAO1 was subjected to transposon (Tn) mutagenesis using the mariner transposon vector, pBT20 [95]. The transposon within pBT20 was conjugally transferred by biparental mating using *E*. *coli* S17-1 λ *pir* into strain PAO1 as previously described [95]. An overnight broth culture of the donor strain (2 ml of stationary phase culture) and 0.5 ml of the recipient strain were clarified by centrifugation and resuspended in 0.5 ml of L-broth. This concentrated suspension was then spotted on the center of a L-broth plate, allowed to dry, and incubated for ~18 hr at 37°C. Mating mixtures were scraped and resuspended in 1 ml of L-broth. Suspensions (300 μl) were spread evenly onto *Pseudomonas* isolation agar (PIA) containing gentamicin (Gm) at 150 μg/ml and incubated at 37°C for 48 hr. The resulting growth was scraped from the plate and resuspended in 2 ml of 0.9% saline and serial dilutions plated onto freshly prepared LBN plates, pH 6.5 containing 15 mM acidified NaNO_2_ (A-NO_2_^-^). Transposon insertion sites were determined through sequencing the flanking region of the transposon by a semi-random PCR method, as described previously [100] using random primer AD2 and transposon-specific primer Gm447, followed by the nested primers Gm464 and AD2 (Table 1). Individual colonies were patched to L-broth plates and L-broth A-NO_2_^-^, pH 6.5 plates containing 15 mM KNO_3_ with either 15 mM or 25 mM NaNO_2_ for selection of sensitive and resistance strains, respectively. Cells were grown aerobically for 24 hr and anaerobically for up to 48 hr. Those organisms that grew on the 25 mM NaNO_2_ plates were considered A-NO_2_^-^ resistant while cells that did not grow on 15 mM NaNO_2_ plates were considered A-NO_2_^—^sensitive. Confirmation of the A-NO_2_^-^ sensitive or resistance phenotype was followed by an A-NO_2_^-^ killing assay by enumeration of remaining CFU after treatment. Briefly, the overnight culture was diluted 1000-fold into L-broth pH 6.5, 50 mM phosphate buffer containing either 15 mM KNO_3_ or 15 mM NaNO_2_, respectively. Cell viability was monitored daily for 3 days. All experiments were performed at least 3 times and reproducible mutants were then assessed for the identity of the specific gene interrupted by the transposon. The genomic DNA was isolated and the identification of the transposon integration site was initiated by semi-random PCR. The resulting PCR amplification products were subjected to DNA sequence analysis at the Cincinnati Children’s Hospital DNA core (Cincinnati, OH).

#### Allelic exchange and sucrose counter-selection for construct of mutants

The strategy for insertional inactivation of *PA* genes (Table 1) was facilitated by gene disruption with an 850-bp Gm^R^ cassette from pUCGM (52), and the gene replacement vector pEX100T-KS (29), the latter of which allowed for selection of double-crossover events within putative recombinants cultured on agar containing 5–6% sucrose. To facilitate construction of an unmarked nonpolar *mucA* deletion mutant, the Gm^R^ cassette from pUCGM was inserted into unique *Sca*I site of pEX100T-KS, creating plasmid pEXΩ100T. Approximately 1 kb of upstream and downstream fragments of the *mucA* gene was PCR amplified, and cloned into the *Hind*III and *Spe*I sites of pEXΩ100T. The resultant plasmid, pEXΩ100TΔ*mucA*, was used to construct a *mucA* deletion mutant (herein termed Δ*mucA*) that contained a downstream constitutive promoter to ensure transcription of the *mucB* gene. All mutants were confirmed by DNA sequencing of amplified PCR products. Two independent *mucA* deletion mutants from the D.J.H. and M.J.S. laboratories were also confirmed by Illumina sequencing (S1 Fig) and the contigs were assembled using the PATRIC alignment program. Other mutants that we suspected to be sensitive to A-NO_2_^-^ from previous studies and our own literature-based hypotheses are also listed in Table 4. Some other mutants listed in Table 4 were also constructed using this method.

#### Planktonic culture measurements of A-NO_2_^-^ sensitivity

Overnight cultures of *PA* and various Tn and/or allelic exchange mutants were 1:100 diluted into either LB broth (pH 6.5) or LBN broth (pH 6.5, the pH of CF airway surface liquid, [9]) supplemented with varying concentrations of NaNO_2_ (0, 5, 10, 15, 20, 25 and 30 mM, hence the term A-NO_2_^-^) and grown anaerobically for 48 hr. Five μl of cells from each culture was serially diluted and spotted onto LB agar plates and incubated aerobically for 24 hr at 37°C. The plates were then scanned for enumeration of CFU. **(ii)** Some strains were also cultured anaerobically for 72 hr in LBN broth (pH 6.5) supplemented with either 0, 20, or 25 mM NaNO_2_ for strain PAO1, Δ*mucA* and *mucA22* mutants, as well as *mucA22 uspK* and *mucA22 fdnG*, respectively. Cultures were processed daily, and serial cell dilutions were spotted onto L-agar plates. Surviving bacteria were enumerated after a 24 hr incubation at 37°C.

#### Anaerobic biofilm A-NO_2_^-^ sensitivity measurements

Bacteria were grown aerobically in LB broth to stationary phase followed by a 1:100 dilution into 3 ml of LBN in confocal friendly glass bottomed chambers (Costar). Static bacterial biofilms were allowed to develop under anaerobic condition as previously described [8]. After 24 hr, biofilms were washed with sterile PBS to remove planktonic cells, and fresh LBN broth (pH 6.5) containing 15 mM KNO_3_ (control), or 15 mM KNO_3_ plus 15 mM NaNO_2_ was added to the bacteria biofilm cultures. The biofilms were then incubated under anaerobic conditions for an additional of 48 hr, washed 2 times with PBS, and stained with Live/Dead BacLight bacterial viability kit (Invitrogen, Eugene, OR). Biofilm images were viewed by confocal laser scanning microscopy using a Zeiss LSM 710 confocal microscope and visualized the live cells in green and the dead cells in red. The excitation and emission wavelengths for green fluorescence (live cells) were 488 nm and 500 nm, while those for red fluorescence (dead cells) were 490 nm and 635 nm, respectively. All biofilm experiments were repeated at least 3 times independently. The live/dead ratio of the biofilms were calculated using imageJ 1.46r software following the guidelines by the University of Chicago Integrated Light Microscopy Core. The results are presented as the differences in the dead/live ratio comparing A-NO_2_^-^treated versus control conditions.

#### Transcriptional profiling using Affymetrix GeneChips of *PA*, *mucA22* and Δ*mucA* strains exposed to A-NO_2_^-^

*PA*, *mucA22* and **Δ***mucA* bacteria were grown anaerobically for 24 hr in LBN, pH 6.5, followed by the addition of 15 mM NaNO_2_ (A-NO_2_^-^) for an additional 20 min. Organisms were then pelleted by centrifugation at 13,000 x *g* for 5 min and the pellets resuspended in RNAlater (Ambion) to prevent bacterial RNA degradation and to stabilize the bacterial mRNA. To assess quantitative gene expression analysis of *PA*, Affymetrix GeneChips were used. RNA from *PA* was isolated by using Qiagen RNeasy columns according to the manufacturer’s protocol for isolation of total RNA. RNA from three independent samples was isolated for hybridization on three independent *Pseudomonas* GeneChips. Once the RNA was eluted from the Qiagen RNeasy column, then RNA was treated with 2 U of DNase I (Ambion) for 15 min at 37°C. The reaction was stopped by the addition of 25 μl of DNase stop solution (50 mM EDTA, 1.5 M sodium acetate and 1% SDS). The DNase I was removed by phenol/chloroform extraction followed by ethanol precipitation. The approximate amount of RNA isolated was quantified using spectrophotometer. To determine the quality of the RNA, samples were analyzed on an Agilent bioanalyzer 2100. The quality of RNA was determined by examining the 16S and 23S rRNA bands on the electrophoretogram that should be at a 1:1 ratio. Ten μg of total RNA was used for cDNA synthesis, fragmentation and labeling according to the Affymetrix GeneChip *PA* Genome Array Expression Analysis pProtocol. Briefly, random hexamers (Invitrogen) were added (final conc. 25 ng/μl) to the 10 μg of total RNA along with *in vitro* transcribed *B*. *subtilis* control spikes (as described in the Affymetrix GeneChip *PA* Genome Array Expression Analysis Protocol). cDNA was synthesized using Superscript II (Invitrogen) according to the manufacturer’s instructions under the following conditions: 25°C for 10 min, 37°C for 60 min, 42°C for 60 min, 70°C for 10 min. RNA was removed by alkaline treatment and subsequent neutralization. The cDNA was purified by a QIAquick PCR purification kit (Qiagen) and eluted in 40 μl of elution buffer (10 mM Tris-HCl, pH 8.5). The cDNA was then fragmented by DNase I (0.6 U per μg cDNA, Amersham) at 37°C for 10 min and end-labeled with Biotin-ddUTP using the Enzo BioArray Terminal Labeling kit (Affymetrix) at 37°C for 60 min. Proper cDNA fragmentation and biotin labeling were determined by gel mobility shift assay using NeutrAvadin (Pierce) and on a 5% polyacrylamide gel stained with SYBR Green I (Roche). The labeled cDNA was hybridized to the Affymetrix *Pseudomonas* GeneChip according to the manufacturer’s protocol. Microarray data was generated using Affymetrix (www.affymetrix.com) protocols as we have done previously [72, 101]. We used Affymetrix data previously obtained from *PA* grown from three independent samples as the control for gene expression comparisons.

**Microarray data analysis.** Microarray data were generated and analyzed using Affymetrix protocols as previously described [102–104]. Probe set summarization (.CHP) files were generated using the Affymetrix MicroArray Suite 5.0 (MAS 5.0) algorithm. The absolute expression transcript levels were normalized for each chip by globally scaling all probe sets to a target signal intensity of 500. Three statistical algorithms (detection, change call, and signal log ratio) were used to identify differential gene expression in experimental and control samples. The decision of a present, absent, or marginal identification for each gene was determined by using MicroArray Suite software (version 5.0; Affymetrix). Those transcripts that received an “absent” designation were removed from further analysis. A *t* test was used to isolate those genes whose transcriptional profile was statistically significant (*P* < 0.05) between the control and experimental conditions. Pair-wise comparisons between the individual experimental and control chips were done by batch analyses using MicroArray Suite to generate a change call and signal log ratio for each transcript. A positive change was defined as a call whereby more than 50% of the transcripts increased or marginally increased for up-regulated genes or decreased or marginally decreased for down-regulated genes. Lastly, the median value of the signal log ratios for each comparison was calculated and only transcripts that had a value greater than or equal to 1 for up-regulated and less than or equal to 1 for down-regulated genes were placed on the final list of transcripts whose profile had changed. The signal-log ratio was converted and expressed as the change (*n*-fold). The microarray data are available on the GEO (Gene Expression Omnibus) website at http://www.ncbi.nlm.nih.gov/projects/geo (GEO accession no. GSE128220).

### Bioinformatic analysis of differential gene expression

The microarray data were analyzed to identify differentially expressed genes (DEGs) between three conditions (anaerobic *PA*, *mucA22* and Δ*mucA* exposed to 15 mM A-NO_2_^-^, pH 6.5 for 20 min) in pairs. The significant DEGs were selected by the criteria that fold change values are larger than two and the *p*-value is lower than 0.05. To elucidate the mechanism underlying A-NO_2_^-^ sensitivity as well as identifying potential interacting partners of MucA, we searched an online *PA* PAO1 protein-protein interaction (PPI) database (http://research.cchmc.org/PPIdatabase) using the query word MucA. This database contains prediction results by a random forest classifier that was trained on eight genomic features (co-essentiality, co-expression, co-functionality, co-localization, domain-domain interaction, co-pathway involvement, transmembrane helices, co-operon and co-gene cluster involvement). The result is a large-scale PPI network in *PA* with significant coverage and high accuracy, i.e., 57,746 potential protein interactions covering 4,256 *PA* PAO1 proteins [86]. Among the DEGs, we selected the potential PPI partners of MucA from the interactome database. We also compared the significant DEGs under each comparison design and looked for overlapping genes, to investigate if the transcriptional profile changes were contributing to the hypersensitivity of A-NO_2_^-^ in *mucA22* mutant bacteria when compared to wild type and Δ*mucA*). The functional annotation clustering of the gene set was conducted by DAVID to see if any functional pathway was related with alternative gene expression. If the overlapping genes were also found in the *mucA22* vs. Δ*mucA* analyses, it indicates that the genes expressed differently should have a tight interaction with MucA. Among the DEGs, we selected the PPI partners for MucA from the interactome database. We also searched the STRING database (https://string-db.org) for predicted MucA interacting partners. The top 20 interactors with high confidence were used for in our analysis. To investigate if the transcriptional profile changes were contributing to the hypersensitivity of A-NO_2_^-^ in *mucA22* comparing to wild type and Δ*mucA*, we also compared the significant DEGs under each comparison design and looked for overlapping genes. The Go function enrichment of the gene set was conducted on the overlapping DEGs and the predicted MucA interactors, in order to see if any functional pathway was related with altered gene expression.

### Identification of anaerobic *S*-nitrosylated proteins in *PA*, *mucA22* and Δ*mucA* strains using the SNOSID technique (SNO Site Identification)

***PA*** PAO1, *mucA*22 and Δ*mucA* were grown in L-broth under aerobic conditions at 37°C for 18 hr. Bacteria were then further diluted 1000-fold in L-broth, pH 6.5 (50 mM potassium phosphate) containing 15 mM KNO_3_ for 24 hr under anaerobic conditions. Bacteria were then exposed to 15 mM NaNO_2_ for 1 hr. The cell pellet was lysed with B-PER plus 0.1 mM EDTA and 0.5 mM PMSF at room temperature for 10 min. Next, identical protein levels were used to evaluate *S*-nitrosylation using the “biotin switch” method as described by Jaffrey and Snyder [105]. Briefly, protein lysates were placed in blocking solution (2.5% SDS and 0.1% methanethiosulfonate, MMTS) prepared in dimethylformamide (DMF) with 9 volumes of HEN buffer (250 mM HEPES-NaOH pH 7.7, 1 mM EDTA and 0.1 mM neocuproine) in the dark at 50°C for 20 min with frequent vortex. The excess MMTS was removed by precipitation with 3 volumes of cold acetone. After centrifugation, the protein pelleted was washed with 70% cold acetone 4 times and then resuspended into HEN buffer containing 1% SDS, 2.5 mg/ml biotin-HPDP and 200 mM sodium ascorbate. The mixtures were incubated in the dark at 25°C for 1 hr with intermittent vortex. The biotinylated nitrosothiols proteins were then precipitated with acetone. Again, after washing 4 times with 70% cold acetone, the protein pelleted was dissolved in 0.1X HEN buffer containing 1% SDS and 3 volumes of neutralization buffer (20 mM HEPES, pH 7.7, 100 mM NaCl, 1 mM EDTA, and 0.5% Triton X-100) was added followed by 50 μl of pre-washed avidin affinity resin. The mixture was then incubated at 4°C for 18 hr. The resin was extensively washed 4 times with 1 ml of washing buffer (20 mM HEPES, pH 7.7, 600 mM NaCl, 1 mM EDTA, and 0.5% Triton X-100) and the resin was fully dried via gentle aspiration with a 28-gauge needle. Bound protein was then eluted with 50 μl of elution buffer (20 mM HEPES, pH 7.7, 100 mM NaCl, 1 mM EDTA, 100 mM 2-mercaptoethanol). The samples were concentrated and desalted using Amicon® Ultra centrifuge filter devices (3 K) and the buffer exchanged 2 times with 50 μl of GE Healthcare Life Sciences DeStreak buffer. The protein concentration was determined using a Pierce 660 nm protein assay and prepared for 2-dimensional gel electrophoresis. Then, 27 μg of purified protein was loaded onto 7 cm IPG strips, pH 3–10 NL (non-linear) and subjected to electrophoresis according to a standardized procedure for the Invitrogen zoom apparatus with no streaking in the first dimension. After running denaturing polyacrylamide gel electrophoresis (SDS-PAGE) for the second dimension, proteins were visualized after silver staining. Gel images were compared using Progenesis SameSpots V3.2 software ((Nonlinear Dynamic, Inc., Durham, NC). The protein spots that revealed significant differences in pairs between *PA* and *mucA22*, *PA* and **Δ***mucA*, or *mucA22* and **Δ***mucA* were then analyzed using matrix-assisted laser desorption ionization-time of flight (MALDI-TOF) mass spectrometry. The proteins were then identified by searching the Swiss-Prot *PA* mass spectrometric database.

### Alginate assays

Selected bacteria were grown in L-broth under aerobic conditions for 24 hr at 37°C with shaking at 250 rpm. The alginate isolation and assay was performed base on the protocol of Ma et. al., [106] with minor modifications. Briefly, the overnight bacterial cultures were mixed 1:1 ratio with PBS and then centrifugation at 16,000 x *g* for 10 min. One volume of 2% cetylpyridinium chloride was added to the supernatant to precipitate the alginate. After centrifugation at the same speed for 5 min, he pelleted was resuspended in 2 volumes of 1 M NaCl and alginate was precipitated using 2 volumes of cold isopropanol. After centrifugation and air drying, the alginate pellet was resuspended in 200 μl of saline. Alginate concentration as mg/ml was calculated using the carbozole assay [107].

### Infection of mouse airways and effects of A-NO_2_^-^ on bacterial viability

Six-week old male Balb/C mice (8 per cohort) were purchased from Harlan Laboratories, Inc. Approximately ~5 x 10^6^ of isogenic *mucA22* or **Δ***mucA* strains were resuspended in 0.9% saline containing purified *PA* alginate at final concentration of 1.1 mg/ml and used 50 μl to inject into mouse tracheas nonsurgically using a 21-gauge ball-end needle to the back of the tongue above the tracheal opening as previously described [108]. The successful delivery of bacteria into the lungs was manifested by a slight gag reflex by the mice exhibited immediately after instillation followed by a pattern of rapid breathing. After 24 hr of incubation, mouse lungs were instilled with 25 μl of 15 mM A-NO_2_^-^ at pH 6.5 (in 0.1 M phosphate buffer) intranasally twice daily. On the fifth day, the mice were sacrificed, and the viable bacteria from serially diluted lung homogenates were enumerated.

### Ethics statement

All animal studies were performed in accordance with the protocols approved by the Animal Care Committee at the University of Illinois at Champagne-Urbana. The animal study was carried out in strict accordance with the recommendations in the Guide for the Care and Use of Laboratory Animals of the National Institutes of Health. The protocol was approved by the Institutional Animal Care and Use Committee (IACUC) at the University of Illinois at Urbana-Champaign (Protocol Number: 15171).

## Supporting information

S1 Fig Two independently generated *mucA* deletions mapped to an identical position on the PAO1 genome. PAO1 ORFs, *PA* PAO1 open reading frames; Red boxes indicate the *mucA* deletion regions. **A.** PAO1 Δ*mucA* (M.J.S. lab), Illumina reads from PAO1 Δ*mucA* aligned to the PAO1 chromosome from the Schurr laboratory; **B.** PAO1 Δ*mucA* (D.J.H.), Illumina reads aligned to PAO1 from PAO1 Δ*mucA* from corresponding author Hassett’s laboratory. **C.** Alignment of wild-type, *mucA22* and Δ*mucA* alleles.
(TIF)

S2 Fig 2-D gel Western blots for SNO proteins in anaerobically grown strains treated with 15 mM A-NO_2_^-^. **A.** PAO1; **B.** *mucA22*; **C.** Δ*mucA*. SNO-proteins were separated using Immobiline DryStrip pH 3–10 NL (non-linear) gels and then silver stained. SNO-proteins revealing differences in signal intensity from each set of protein spots were extracted from the gels and identified by mass spectrometry. The identification of each circled protein is listed in Table 3 with the fold up or down values given.
(TIF)

S1 Table Fold change in gene expression of anaerobic PAO1 vs. *mucA22* upon exposure to 15 mM A-NO_2_^-^. The change up/down are values in the *mucA22* mutant relative to that of strain PAO1. IG, intragenic region.
(DOCX)

S2 Table Fold change in gene expression of anaerobic PAO1 vs. Δ*mucA* upon exposure to 20 mM A-NO_2_^-^. The change up/down are values in the Δ*mucA* mutant relative to that of strain PAO1.
(DOCX)

S3 Table Fold change in gene expression of anaerobic *mucA22* vs. Δ*mucA* bacteria upon exposure to 15 mM A-NO_2_^-^. The change up/down are values in the Δ*mucA* mutant relative to that of strain *mucA22*.
(DOCX)

S4 Table Inability of the FRD1 *norCB* genes to complement an anaerobic growth defect of a PAO1 *norCB* mutant. (DOCX)

S5 Table Overlapping differentially expressed genes (DEGs) in 3 comparisons. (DOCX)

S6 Table Predicted interactors of MucA. Differentially expressed genes (DEGs) are in red.
(DOCX)

S7 Table GO function enrichment of overlapping DEGs in *mucA22* vs Δ*mucA* and PAO1 vs *mucA22*, and predicted MucA interactors. (DOCX)
