# Supplementary figures and images for: The anti-sigma factor MucA of Pseudomonas aeruginosa: Dramatic differences of a mucA22 vs. a ΔmucA mutant in anaerobic acidified nitrite sensitivity of planktonic and biofilm bacteria in vitro and during chronic murine lung infection
Source: PLoS One. 2019 Jun 3;14(6):e0216401. doi: 10.1371/journal.pone.0216401 (PMC6546240; doi:10.1371/journal.pone.0216401)

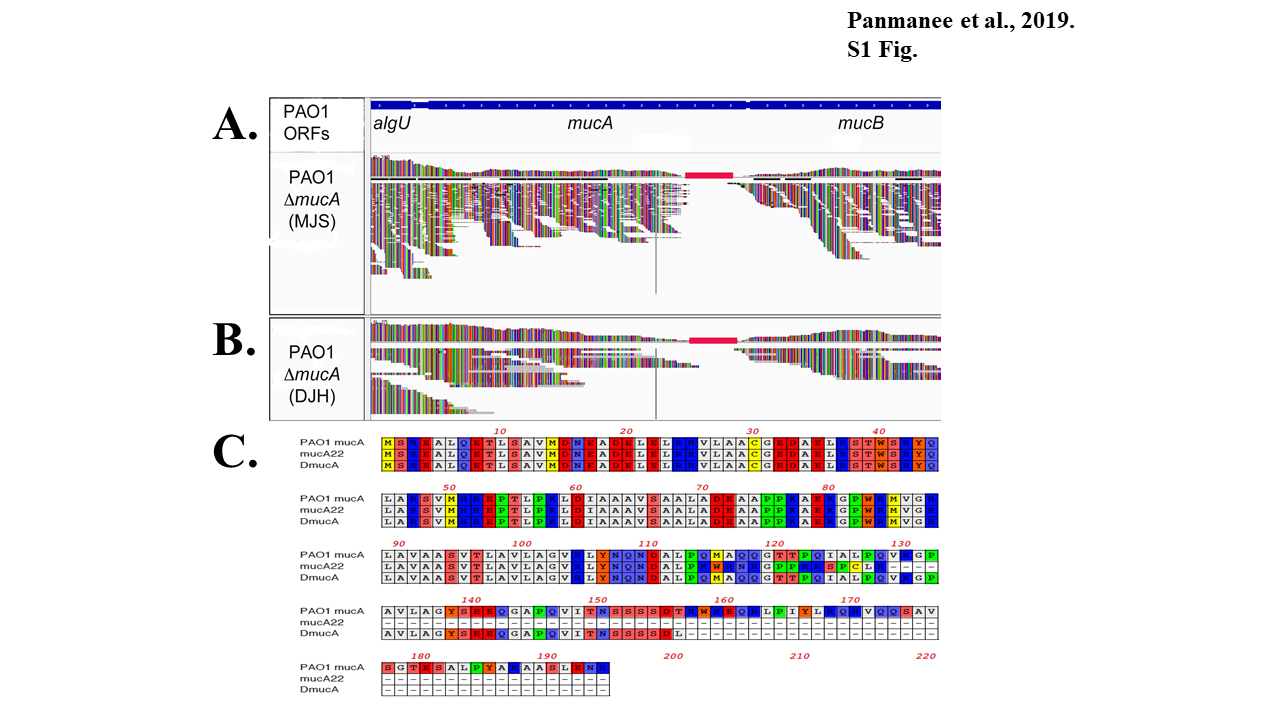

Supplement: S1 Fig — PAO1 ORFs, PA PAO1 open reading frames; Red boxes indicate the mucA deletion regions. A. PAO1 ΔmucA (M.J.S. lab), Illumina reads from PAO1 ΔmucA aligned to the PAO1 chromosome from the Schurr laboratory; B. PAO1 ΔmucA (D.J.H.), Illumina reads aligned to PAO1 from PAO1 ΔmucA from corresponding author Hassett’s laboratory. C. Alignment of wild-type, mucA22 and ΔmucA alleles. (TIF) [file pone.0216401.s001.TIF]

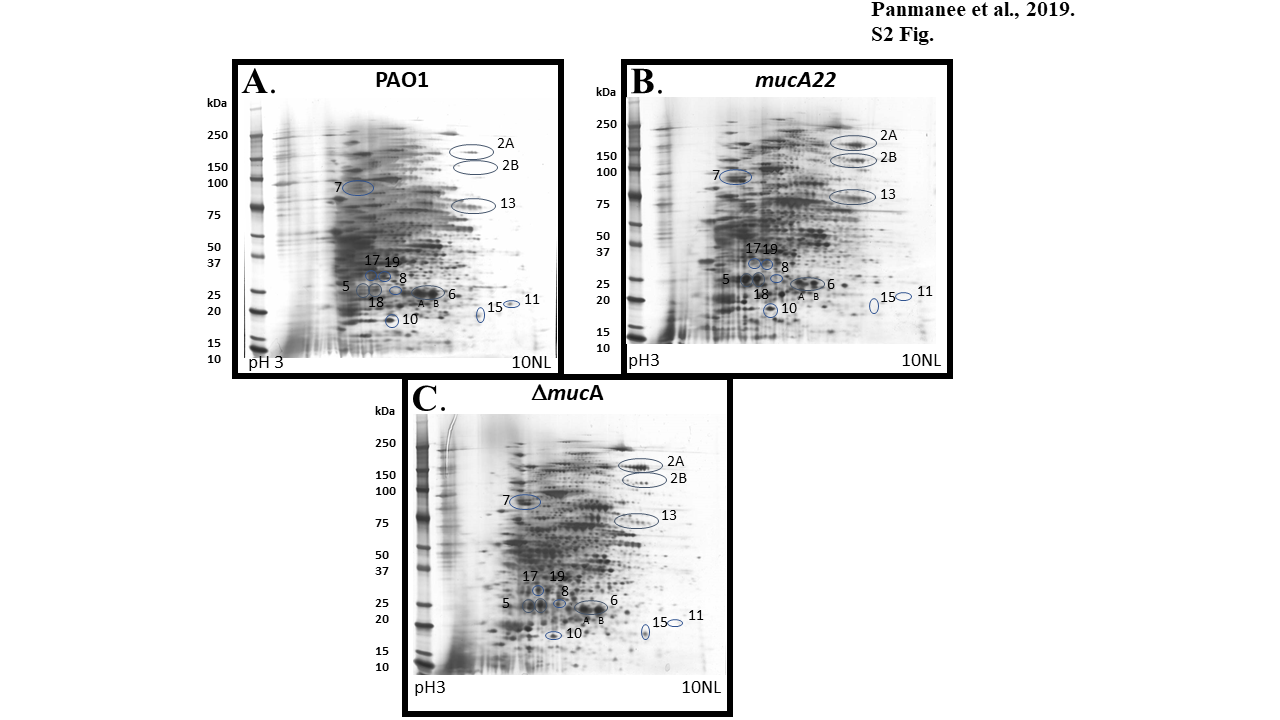

Supplement: S2 Fig — A. PAO1; B. mucA22; C. ΔmucA. SNO-proteins were separated using Immobiline DryStrip pH 3–10 NL (non-linear) gels and then silver stained. SNO-proteins revealing differences in signal intensity from each set of protein spots were extracted from the gels and identified by mass spectrometry. The identification of each circled protein is listed in Table 3 with the fold up or down values given. (TIF) [file pone.0216401.s002.TIF]
