# Supplementary material for: The anti-sigma factor MucA of Pseudomonas aeruginosa: Dramatic differences of a mucA22 vs. a ΔmucA mutant in anaerobic acidified nitrite sensitivity of planktonic and biofilm bacteria in vitro and during chronic murine lung infection
Source: PLoS One. 2019 Jun 3;14(6):e0216401. doi: 10.1371/journal.pone.0216401 (PMC6546240; doi:10.1371/journal.pone.0216401)
Supplement: S1 Table — The change up/down are values in the mucA22 mutant relative to that of strain PAO1. IG, intragenic region. (DOCX) [file pone.0216401.s003.docx]

| **Probe Set ID** | **Gene Symbol** | **Fold Change** | **Change** | **Probe Set ID** | **Gene Symbol** | **Fold Change** | **Change** |
| --- | --- | --- | --- | --- | --- | --- | --- |
| PA4131 |  | 52.763 | Down | PA5383 |  | 11.241 | Up |
| PA4132 |  | 20.652 | Down | PA0284 |  | 11.175 | Up |
| PA4130 |  | 18.958 | Down | PA0283 | *sbp* | 10.783 | Up |
| PA0524 | *norB* | 12.862 | Down | PA3931 |  | 9.338 | Up |
| PA0432 | *sahH* | 7.349 | Down | PA0201 |  | 9.147 | Up |
| PA4129 |  | 6.911 | Down | PA3450 |  | 7.141 | Up |
| PA0431 |  | 6.551 | Down | PA2659 |  | 6.817 | Up |
| PA0547 |  | 4.573 | Down | PA0795 | *prpC* | 6.465 | Up |
| PA5304 | *dadA* | 4.124 | Down | PA0281 | *cysW* | 6.143 | Up |
| PA0546 | *metK* | 3.869 | Down | PA0320 |  | 5.955 | Up |
| PA5250 |  | 3.646 | Down | PA2658 |  | 5.599 | Up |
| PA4033 |  | 3.529 | Down | PA0796 | *prpB* | 5.104 | Up |
| PA4933 |  | 3.439 | Down | PA3446 |  | 4.883 | Up |
| PA0179 |  | 3.335 | Down | PA1136 |  | 4.654 | Up |
| PA5251 |  | 3.193 | Down | PA3551 | *algA* | 4.121 | Up |
| PA0548 | *tktA* | 3.059 | Down | PA0671 |  | 3.968 | Up |
| PA5252 |  | 3.039 | Down | PA2663 |  | 3.864 | Up |
| PA4630 |  | 3.026 | Down | PA0797 |  | 3.848 | Up |
| PA4932 | *rplI* | 2.916 | Down | PA1135 |  | 3.606 | Up |
| PA4636 |  | 2.893 | Down | PA2662 |  | 3.566 | Up |
| PA4030 |  | 2.871 | Down | PA2934 |  | 3.358 | Up |
| PA0519 | *nirS* | 2.814 | Down | PA1137 |  | 3.319 | Up |
| PA0517 | *nirC* | 2.751 | Down | PA0793 |  | 3.208 | Up |
| PA4935 | *rpsF* | 2.73 | Down | PA1239 |  | 3.155 | Up |
| PA0549 |  | 2.711 | Down | PA3118 | *leuB* | 3.026 | Up |
| PA3787 |  | 2.524 | Down | PA2015 | *gnyD* | 2.984 | Up |
| PA3748 |  | 2.489 | Down | PA3195 | *gapA* | 2.981 | Up |
| PA5203 | *gshA* | 2.488 | Down | PA3472 |  | 2.96 | Up |
| PA0396 | *pilU* | 2.399 | Down | PA0280 | *cysA* | 2.942 | Up |
| PA4768 | *smpB* | 2.391 | Down | PA3932 |  | 2.928 | Up |
| PA1132 |  | 2.365 | Down | PA2657 |  | 2.837 | Up |
| PA5163 | *rmlA* | 2.365 | Down | PA1587 | *lpdG* | 2.834 | Up |
| PA0293 | *aguB* | 2.363 | Down | PA3119 |  | 2.727 | Up |
| PA4853 | *Fis* | 2.361 | Down | ig_326671_327284_at | | 2.687 | Up |
| PA5162 | *rmlD* | 2.297 | Down | PA1865 |  | 2.663 | Up |
| PA3747 |  | 2.249 | Down | PA4443 | *cysD* | 2.656 | Up |
| PA4234 | *uvrA* | 2.243 | Down | PA0807 |  | 2.507 | Up |
| PA4059 |  | 2.228 | Down | PA0794 |  | 2.457 | Up |
| PA4915 |  | 2.212 | Down | PA2287 |  | 2.453 | Up |
| PA1423 |  | 2.195 | Down | PA0792 | *prpD* | 2.356 | Up |
| PA0045 |  | 2.191 | Down | PA0291 | *oprE* | 2.312 | Up |
| PA0126 |  | 2.142 | Down | PA2288 |  | 2.309 | Up |
| PA4971 | *Asp* | 2.118 | Down | PA3008 |  | 2.208 | Up |
| PA4769 |  | 2.117 | Down | PA2644 | *nuoI* | 2.185 | Up |
| PA5483 | *algB* | 2.111 | Down | PA3120 | *leuD* | 2.168 | Up |
| PA5147 | *mutY* | 2.074 | Down | PA2599 |  | 2.148 | Up |
| PA2840 |  | 2.065 | Down | PA0425 | *mexA* | 2.124 | Up |
| PA4839 | *speA* | 2.019 | Down | PA1805 | *ppiD* | 2.036 | Up |
| PA5125 | *ntrC* | 2.009 | Down |  |  |  |  |
