# Supplementary material for: The anti-sigma factor MucA of Pseudomonas aeruginosa: Dramatic differences of a mucA22 vs. a ΔmucA mutant in anaerobic acidified nitrite sensitivity of planktonic and biofilm bacteria in vitro and during chronic murine lung infection
Source: PLoS One. 2019 Jun 3;14(6):e0216401. doi: 10.1371/journal.pone.0216401 (PMC6546240; doi:10.1371/journal.pone.0216401)
Supplement: S2 Table — The change up/down are values in the ΔmucA mutant relative to that of strain PAO1. (DOCX) [file pone.0216401.s004.docx]

| **Probe Set ID** | | **Gene Symbol** | | **Fold Change** | | **Change** | | **Probe Set ID** | **Gene Symbol** | | **Fold Change** | | **Change** | |
| --- | --- | --- | --- | --- | --- | --- | --- | --- | --- | --- | --- | --- | --- | --- |
| PA0045 | |  | | 2.733 | | down | | PA1431 | *rsaL* | | 11.518 | | up | |
| PA0179 | |  | | 2.711 | | down | | PA1432 | *lasI* | | 11.308 | | up | |
| PA0286 | |  | | 2.262 | | down | | PA0807 |  | | 10.122 | | up | |
| PA0432 | | *sahH* | | 3.959 | | down | | PA3181 |  | | 8.462 | | up | |
| PA0546 | | *metK* | | 7.982 | | down | | PA1746 |  | | 8.333 | | up | |
| PA0547 | |  | | 8.878 | | down | | PA0518 | *nirM* | | 7.107 | | up | |
| PA0764 | | *mucB* | | 10.74 | | down | | PA2663 |  | | 7.022 | | up | |
| PA0765 | | *mucC* | | 5.066 | | down | | PA2662 |  | | 6.807 | | up | |
| PA0766 | | *mucD* | | 4.451 | | down | | PA3392 | *nosZ* | | 6.55 | | up | |
| PA0933 | | *ygcA* | | 2.136 | | down | | PA0670 |  | | 6.453 | | up | |
| PA0938 | |  | | 2.15 | | down | | PA0671 |  | | 6.435 | | up | |
| PA1132 | |  | | 2.585 | | down | | PA0519 | *nirS* | | 6.014 | | up | |
| PA1423 | |  | | 3.252 | | down | | PA0520 | *nirQ* | | 5.716 | | up | |
| PA1541 | |  | | 6.462 | | down | | PA0517 | *nirC* | | 5.698 | | up | |
| PA2277 | | *arsR* | | 2.571 | | down | | PA2664 | *Fhp* | | 5.084 | | up | |
| PA2290 | | *Gcd* | | 2.07 | | down | | PA4810 | *fdnI* | | 5.084 | | up | |
| PA2788 | |  | | 2.151 | | down | | PA0910 |  | | 5.064 | | up | |
| PA2830 | | *htpX* | | 2.286 | | down | | PA1048 |  | | 4.998 | | up | |
| PA2869 | |  | | 3.165 | | down | | PA0069 |  | | 4.874 | | up | |
| PA3011 | | *topA* | | 2.265 | | down | | PA4812 | *fdnG* | | 4.746 | | up | |
| PA3066 | |  | | 2.653 | | down | | PA0516 | *nirF* | | 4.71 | | up | |
| PA3067 | |  | | 4.728 | | down | | PA3972 |  | | 4.603 | | up | |
| PA3747 | |  | | 2.405 | | down | | PA0515 |  | | 4.212 | | up | |
| PA4006 | | *nadD* | | 2.408 | | down | | PA0911 |  | | 4.081 | | up | |
| PA4007 | | *proA* | | 2.021 | | down | | PA2706 |  | | 3.997 | | up | |
| PA4033 | |  | | 3.066 | | down | | PA0558 |  | | 3.94 | | up | |
| PA4045 | |  | | 2.099 | | down | | PA0526 |  | | 3.894 | | up | |
| PA4046 | |  | | 2.182 | | down | | PA3551 | *algA* | | 3.813 | | up | |
| PA4061 | |  | | 2.074 | | down | | PA2643 | *nuoH* | | 3.676 | | up | |
| PA4499 | |  | | 3.036 | | down | | PA3973 |  | | 3.676 | | up | |
| PA4625 | |  | | 2.706 | | down | | PA3575 |  | | 3.619 | | up | |
| PA4630 | |  | | 2.865 | | down | | PA2646 | *nuoK* | | 3.597 | | up | |
| PA4919 | | *pncB1* | | 2.464 | | down | | PA2705 |  | | 3.576 | | up | |
| PA4971 | | *Asp* | | 2.047 | | down | | PA2445 | *gcvP2* | | 3.569 | | up | |
| PA5203 | | *gshA* | | 3.09 | | down | | PA5479 | *gltP* | | 3.559 | | up | |
| PA5250 | |  | | 3.614 | | down | | PA2642 | *nuoG* | | 3.541 | | up | |
| PA5251 | |  | | 3.253 | | down | | PA2667 |  | | 3.527 | | up | |
| PA5252 | |  | | 2.72 | | down | | PA2381 |  | | 3.503 | | up | |
| PA5483 | | *algB* | | 2.51 | | down | | PA0224 |  | | 3.493 | | up | |
| PA5557 | | *atpH* | | 2.072 | | down | | PA3472 |  | | 3.424 | | up | |
| PA5564 | | *gidB* | | 2 | | down | | PA0161 |  | | 3.321 | | up | |
| PA5565 | | *gidA* | | 2.122 | | down | | PA3971 |  | | 3.304 | | up | |
|  | |  | |  | |  | | PA2644 | *nuoI* | | 3.204 | | up | |
|  | |  | |  | |  | | PA2641 | *nuoF* | | 3.187 | | up | |
|  | |  | |  | |  | | PA0731 |  | | 3.172 | | up | |
|  | |  | |  | |  | | PA2718 |  | | 3.153 | | up | |
|  | |  | |  | |  | | PA2846 |  | | 3.019 | | up | |
|  | |  | |  | |  | | PA3620 | *mutS* | | 2.994 | | up | |
|  | |  | |  | |  | | PA0669 |  | | 2.979 | | up | |
|  | |  | |  | |  | | PA0413 | *chpA* | | 2.975 | | up | |
|  | |  | |  | |  | | PA2640 | *nuoE* | | 2.938 | | up | |
|  | |  | |  | |  | | PA3859 |  | | 2.934 | | up | |
|  | |  | |  | |  | | PA5496 |  | | 2.852 | | up | |
|  | |  | |  | |  | | PA1587 | *lpdG* | | 2.807 | | up | |
|  | |  | |  | |  | | PA1589 | *sucD* | | 2.684 | | up | |
|  | |  | |  | |  | | PA5429 | *aspA* | | 2.681 | | up | |
|  | |  | |  | |  | | PA3471 |  | | 2.676 | | up | |
|  | |  | |  | |  | | PA2639 | *nuoD* | | 2.619 | | up | |
|  | |  | |  | |  | | PA2648 | *nuoM* | | 2.608 | | up | |
|  | |  | |  | |  | | PA3567 |  | | 2.598 | | up | |
|  | |  | |  | |  | | PA2948 | *cobM* | | 2.545 | | up | |
|  | |  | |  | |  | | PA1588 | *sucC* | | 2.533 | | up | |
|  | |  | |  | |  | | PA1865 |  | | 2.533 | | up | |
|  | |  | |  | |  | | PA2796 | *Tal* | | 2.512 | | up | |
|  | |  | |  | |  | | PA2658 |  | | 2.401 | | up | |
|  | |  | |  | |  | | PA0083 |  | | 2.386 | | up | |
|  | |  | |  | |  | | PA2946 |  | | 2.379 | | up | |
|  | |  | |  | |  | | PA4180 |  | | 2.343 | | up | |
|  | |  | |  | |  | | PA0510 |  | | 2.332 | | up | |
|  | |  | |  | |  | | PA4440 |  | | 2.321 | | up | |
|  | |  | |  | |  | | PA0124 |  | | 2.298 | | up | |
|  | |  | |  | |  | | PA0410 | *pilI* | | 2.145 | | up | |
|  | |  | |  | |  | | PA3013 | *foaB* | | 2.143 | | up | |
|  | |  | |  | |  | | PA0906 |  | | 2.138 | | up | |
|  | |  | |  | |  | | PA0082 |  | | 2.116 | | up | |
|  | |  | |  | |  | | PA4803 |  | | 2.099 | | up | |
|  | |  | |  | |  | | PA0412 | *pilK* | | 2.043 | | up | |
|  | |  | |  | |  | | PA0353 | *ilvD* | | 2.041 | | up | |
|  | |  | |  | |  | | PA4493 |  | | 2.035 | | up | |
|  | |  | |  | |  | | PA3012 |  | | 2.026 | | up | |
|  | |  | |  | |  | | PA4068 |  | | 2.02 | | up | |
| **Probe Set ID** | | **Gene Symbol** | | **Fold Change** | | **Change** | **Probe Set ID** | | **Gene Symbol** | | **Fold Change** | | **Change** |  |
| PA0045 | |  | | 2.733 | | down | PA1431 | | *rsaL* | | 11.518 | | up |  |
| PA0179 | |  | | 2.711 | | down | PA1432 | | *lasI* | | 11.308 | | up |  |
| PA0286 | |  | | 2.262 | | down | PA0807 | |  | | 10.122 | | up |  |
| PA0432 | | *sahH* | | 3.959 | | down | PA3181 | |  | | 8.462 | | up |  |
| PA0546 | | *metK* | | 7.982 | | down | PA1746 | |  | | 8.333 | | up |  |
| PA0547 | |  | | 8.878 | | down | PA0518 | | *nirM* | | 7.107 | | up |  |
| PA0764 | | *mucB* | | 10.74 | | down | PA2663 | |  | | 7.022 | | up |  |
| PA0765 | | *mucC* | | 5.066 | | down | PA2662 | |  | | 6.807 | | up |  |
| PA0766 | | *mucD* | | 4.451 | | down | PA3392 | | *nosZ* | | 6.55 | | up |  |
| PA0933 | | *ygcA* | | 2.136 | | down | PA0670 | |  | | 6.453 | | up |  |
| PA0938 | |  | | 2.15 | | down | PA0671 | |  | | 6.435 | | up |  |
| PA1132 | |  | | 2.585 | | down | PA0519 | | *nirS* | | 6.014 | | up |  |
| PA1423 | |  | | 3.252 | | down | PA0520 | | *nirQ* | | 5.716 | | up |  |
| PA1541 | |  | | 6.462 | | down | PA0517 | | *nirC* | | 5.698 | | up |  |
| PA2277 | | *arsR* | | 2.571 | | down | PA2664 | | *fhp* | | 5.084 | | up |  |
| PA2290 | | *gcd* | | 2.07 | | down | PA4810 | | *fdnI* | | 5.084 | | up |  |
| PA2788 | |  | | 2.151 | | down | PA0910 | |  | | 5.064 | | up |  |
| PA2830 | | *htpX* | | 2.286 | | down | PA1048 | |  | | 4.998 | | up |  |
| PA2869 | |  | | 3.165 | | down | PA0069 | |  | | 4.874 | | up |  |
| PA3011 | | *topA* | | 2.265 | | down | PA4812 | | *fdnG* | | 4.746 | | up |  |
| PA3066 | |  | | 2.653 | | down | PA0516 | | *nirF* | | 4.71 | | up |  |
| PA3067 | |  | | 4.728 | | down | PA3972 | |  | | 4.603 | | up |  |
| PA3747 | |  | | 2.405 | | down | PA0515 | |  | | 4.212 | | up |  |
| PA4006 | | *nadD* | | 2.408 | | down | PA0911 | |  | | 4.081 | | up |  |
| PA4007 | | *proA* | | 2.021 | | down | PA2706 | |  | | 3.997 | | up |  |
| PA4033 | |  | | 3.066 | | down | PA0558 | |  | | 3.94 | | up |  |
| PA4045 | |  | | 2.099 | | down | PA0526 | |  | | 3.894 | | up |  |
| PA4046 | |  | | 2.182 | | down | PA3551 | | *algA* | | 3.813 | | up |  |
| PA4061 | |  | | 2.074 | | down | PA2643 | | *nuoH* | | 3.676 | | up |  |
| PA4499 | |  | | 3.036 | | down | PA3973 | |  | | 3.676 | | up |  |
| PA4625 | |  | | 2.706 | | down | PA3575 | |  | | 3.619 | | up |  |
| PA4630 | |  | | 2.865 | | down | PA2646 | | *nuoK* | | 3.597 | | up |  |
| PA4919 | | *pncB1* | | 2.464 | | down | PA2705 | |  | | 3.576 | | up |  |
| PA4971 | | *asp* | | 2.047 | | down | PA2445 | | *gcvP2* | | 3.569 | | up |  |
| PA5203 | | *gshA* | | 3.09 | | down | PA5479 | | *gltP* | | 3.559 | | up |  |
| PA5250 | |  | | 3.614 | | down | PA2642 | | *nuoG* | | 3.541 | | up |  |
| PA5251 | |  | | 3.253 | | down | PA2667 | |  | | 3.527 | | up |  |
| PA5252 | |  | | 2.72 | | down | PA2381 | |  | | 3.503 | | up |  |
| PA5483 | | *algB* | | 2.51 | | down | PA0224 | |  | | 3.493 | | up |  |
| PA5557 | | *atpH* | | 2.072 | | down | PA3472 | |  | | 3.424 | | up |  |
| PA5564 | | *gidB* | | 2 | | down | PA0161 | |  | | 3.321 | | up |  |
| PA5565 | | *gidA* | | 2.122 | | down | PA3971 | |  | | 3.304 | | up |  |
|  | |  | |  | |  | PA2644 | | *nuoI* | | 3.204 | | up |  |
|  | |  | |  | |  | PA2641 | | *nuoF* | | 3.187 | | up |  |
|  | |  | |  | |  | PA0731 | |  | | 3.172 | | up |  |
|  | |  | |  | |  | PA2718 | |  | | 3.153 | | up |  |
|  | |  | |  | |  | PA2846 | |  | | 3.019 | | up |  |
|  | |  | |  | |  | PA3620 | | *mutS* | | 2.994 | | up |  |
|  | |  | |  | |  | PA0669 | |  | | 2.979 | | up |  |
|  | |  | |  | |  | PA0413 | | *chpA* | | 2.975 | | up |  |
|  | |  | |  | |  | PA2640 | | *nuoE* | | 2.938 | | up |  |
|  | |  | |  | |  | PA3859 | |  | | 2.934 | | up |  |
|  | |  | |  | |  | PA5496 | |  | | 2.852 | | up |  |
|  | |  | |  | |  | PA1587 | | *lpdG* | | 2.807 | | up |  |
|  | |  | |  | |  | PA1589 | | *sucD* | | 2.684 | | up |  |
|  | |  | |  | |  | PA5429 | | *aspA* | | 2.681 | | up |  |
|  | |  | |  | |  | PA3471 | |  | | 2.676 | | up |  |
|  | |  | |  | |  | PA2639 | | *nuoD* | | 2.619 | | up |  |
|  | |  | |  | |  | PA2648 | | *nuoM* | | 2.608 | | up |  |
|  | |  | |  | |  | PA3567 | |  | | 2.598 | | up |  |
|  | |  | |  | |  | PA2948 | | *cobM* | | 2.545 | | up |  |
|  | |  | |  | |  | PA1588 | | *sucC* | | 2.533 | | up |  |
|  | |  | |  | |  | PA1865 | |  | | 2.533 | | up |  |
|  | |  | |  | |  | PA2796 | | *tal* | | 2.512 | | up |  |
|  | |  | |  | |  | PA2658 | |  | | 2.401 | | up |  |
|  | |  | |  | |  | PA0083 | |  | | 2.386 | | up |  |
|  | |  | |  | |  | PA2946 | |  | | 2.379 | | up |  |
|  | |  | |  | |  | PA4180 | |  | | 2.343 | | up |  |
|  | |  | |  | |  | PA0510 | |  | | 2.332 | | up |  |
|  | |  | |  | |  | PA4440 | |  | | 2.321 | | up |  |
|  | |  | |  | |  | PA0124 | |  | | 2.298 | | up |  |
|  | |  | |  | |  | PA0410 | | *pilI* | | 2.145 | | up |  |
|  | |  | |  | |  | PA3013 | | *foaB* | | 2.143 | | up |  |
|  | |  | |  | |  | PA0906 | |  | | 2.138 | | up |  |
|  | |  | |  | |  | PA0082 | |  | | 2.116 | | up |  |
|  | |  | |  | |  | PA4803 | |  | | 2.099 | | up |  |
|  | |  | |  | |  | PA0412 | | *pilK* | | 2.043 | | up |  |
|  | |  | |  | |  | PA0353 | | *ilvD* | | 2.041 | | up |  |
|  | |  | |  | |  | PA4493 | |  | | 2.035 | | up |  |
|  | |  | |  | |  | PA3012 | |  | | 2.026 | | up |  |
|  | |  | |  | |  | PA4068 | |  | | 2.02 | | up |  |
