# Supplementary material for: The anti-sigma factor MucA of Pseudomonas aeruginosa: Dramatic differences of a mucA22 vs. a ΔmucA mutant in anaerobic acidified nitrite sensitivity of planktonic and biofilm bacteria in vitro and during chronic murine lung infection
Source: PLoS One. 2019 Jun 3;14(6):e0216401. doi: 10.1371/journal.pone.0216401 (PMC6546240; doi:10.1371/journal.pone.0216401)
Supplement: S3 Table — The change up/down are values in the ΔmucA mutant relative to that of strain mucA22. (DOCX) [file pone.0216401.s005.docx]

| **Probe Set ID** | **Gene Symbol** | **Fold Change** | **Change** | **Probe Set ID** | **Gene Symbol** | **Fold Change** | **Change** |
| --- | --- | --- | --- | --- | --- | --- | --- |
| PA0284 |  | 21.026 | Down | PA0524 | *norB* | 68.397 | up |
| PA0283 | *sbp* | 19.074 | Down | PA0523 | *norC* | 42.779 | up |
| PA4156 |  | 14.019 | Down | PA0519 | *nirS* | 16.926 | up |
| PA3931 |  | 13.068 | Down | PA0517 | *nirC* | 15.68 | up |
| PA3450 |  | 9.23 | Down | PA1432 | *lasI* | 14.758 | up |
| PA0764 | *mucB* | 8.594 | Down | PA0518 | *nirM* | 14.016 | up |
| PA0281 | *cysW* | 8.017 | Down | PA0525 |  | 12.069 | up |
| PA0766 | *mucD* | 5.039 | Down | PA0515 |  | 10.873 | up |
| PA3446 |  | 4.798 | Down | PA0520 | *nirQ* | 9.466 | up |
| PA4155 |  | 4.751 | Down | PA0516 | *nirF* | 9.405 | up |
| PA0201 |  | 4.666 | Down | PA3880 |  | 6.491 | up |
| PA4158 | *fepC* | 4.391 | Down | PA4810 | *fdnI* | 4.81 | up |
| PA4443 | *cysD* | 3.353 | Down | PA0807 |  | 4.036 | up |
| PA3530 |  | 3.166 | Down | PA5429 | *aspA* | 3.789 | up |
| PA0280 | *cysA* | 2.936 | Down | PA3630 |  | 2.912 | up |
| PA4161 | *fepG* | 2.728 | Down | PA3971 |  | 2.775 | up |
| PA1838 | *cysI* | 2.595 | Down | PA3632 |  | 2.734 | up |
| PA2830 | *htpX* | 2.287 | Down | PA0510 |  | 2.561 | up |
| PA1059 |  | 2.285 | Down | PA1083 | *flgH* | 2.123 | up |
| PA2687 | *pfeS* | 2.268 | Down | PA0025 | *aroE* | 2.119 | up |
| PA4896 |  | 2.236 | Down | PA0396 | *pilU* | 2.095 | up |
| PA1837 |  | 2.116 | Down |  |  |  |  |
| PA2599 |  | 2.042 | Down |  |  |  |  |
| PA3413 |  | 2.023 | Down |  |  |  |  |
