# Supplementary material for: The anti-sigma factor MucA of Pseudomonas aeruginosa: Dramatic differences of a mucA22 vs. a ΔmucA mutant in anaerobic acidified nitrite sensitivity of planktonic and biofilm bacteria in vitro and during chronic murine lung infection
Source: PLoS One. 2019 Jun 3;14(6):e0216401. doi: 10.1371/journal.pone.0216401 (PMC6546240; doi:10.1371/journal.pone.0216401)
Supplement: S4 Table — (DOCX) [file pone.0216401.s006.docx]

| Strain | O.D. 600 nm |
| --- | --- |
| PAO1 | 0.287+/-0.021 |
| PAO1 *norCB* | 0.097+/-0.002 |
| PAO1 *norCB*  *attB*-PAO1 *norCB* | 0.206+/-0.025 |
