# Supplementary material for: The anti-sigma factor MucA of Pseudomonas aeruginosa: Dramatic differences of a mucA22 vs. a ΔmucA mutant in anaerobic acidified nitrite sensitivity of planktonic and biofilm bacteria in vitro and during chronic murine lung infection
Source: PLoS One. 2019 Jun 3;14(6):e0216401. doi: 10.1371/journal.pone.0216401 (PMC6546240; doi:10.1371/journal.pone.0216401)
Supplement: S5 Table — (DOCX) [file pone.0216401.s007.docx]

| ***mucA22* vs Δ*mucA*** | ***mucA22* vs Δ*mucA*** | **PAO1 vs *mucA22*** | **Overlap of three comparisons** |
| --- | --- | --- | --- |
| **And** | **and** | **and** |  |
| **PAO1 vs *mucA22*** | **PAO1 vs Δ*mucA*** | **PAO1 vs Δ*mucA*** |  |
| PA0201 | PA0510 | PA0045 | PA0517 *nirC* |
| PA0280 *cysA* | PA0515 | PA0179 | PA0519 *nirS* |
| PA0281 *cysW* | PA0516 *nirF* | PA0432 *sahH* | PA0807 |
| PA0283 *sbp* | PA0517 *nirC* | PA0517 *nirC* |  |
| PA0284 | PA0518 *nirM* | PA0519 *nirS* |  |
| PA0369 *pilU* | PA0519 *nirS* | PA0546 *metK* |  |
| PA0517 *nirC* | PA0520 *nirQ* | PA0547 |  |
| PA0519 *nirS* | PA0764 *mucB* | PA0671 |  |
| PA0524 *norB* | PA0766 *nucD* | PA0807 |  |
| PA0807 | PA0807 | PA1132 |  |
| PA2599 | PA1432 *lasI* | PA1423 |  |
| PA3446 | PA2830 *htpX* | PA1587 *lpdG* |  |
| PA3450 | PA3971 | PA1865 |  |
| PA3931 | PA4810 *fdnL* | PA2644 *nuoL* |  |
| PA4443 *cysD* | PA5429 *aspA* | PA2658 |  |
|  |  | PA2662 |  |
|  |  | PA2663 |  |
|  |  | PA3472 |  |
|  |  | PA3551 *algA* |  |
|  |  | PA3747 |  |
|  |  | PA4033 |  |
|  |  | PA4630 |  |
|  |  | PA4971 |  |
|  |  | PA5203 *gshA* |  |
|  |  | PA5250 |  |
|  |  | PA5251 |  |
|  |  | PA5252 |  |
|  |  | PA5483 *algB* |  |
