# Supplementary material for: The anti-sigma factor MucA of Pseudomonas aeruginosa: Dramatic differences of a mucA22 vs. a ΔmucA mutant in anaerobic acidified nitrite sensitivity of planktonic and biofilm bacteria in vitro and during chronic murine lung infection
Source: PLoS One. 2019 Jun 3;14(6):e0216401. doi: 10.1371/journal.pone.0216401 (PMC6546240; doi:10.1371/journal.pone.0216401)
Supplement: S6 Table — Differentially expressed genes (DEGs) are in red. (DOCX) [file pone.0216401.s008.docx]

| **ID** | **Name** | **Confidence** | **Source** |
| --- | --- | --- | --- |
| PA0762 | *algU* | 1 | Both |
| PA4446 | *algW* | 1 | Both |
| **PA0764** | ***mucB*** | **0.97** | **both** |
| **PA0765** | ***mucC*** | **0.97** | **Both** |
| **PA0766** | ***mucD*** | **0.97** | **Both** |
| PA0610 | *prtN* | 0.57 | PPI database |
| **PA0517** | ***nirC*** | **0.5** | **PPI database** |
| PA3644 | *lpxA* | 1 | PPI database |
| **PA3551** | ***algA*** | **0.83** | **PPI database** |
| PA5452 | *wbpW* | 0.83 | PPI database |
| PA4996 | *hldE* | 0.78 | PPI database |
| PA4201 | *ddlA* | 0.72 | PPI database |
| PA3666 | *dapD* | 0.69 | PPI database |
| PA4481 | *mreB* | 0.57 | PPI database |
| PA2622 | *cspD* | 0.54 | PPI database |
| PA4406 | *lpxC* | 0.54 | PPI database |
| PA4726 | *cbrB* | 0.52 | PPI database |
| PA0675 | *vreI* | 0.876 | STRING |
| PA0472 | PA0472 | 0.86 | STRING |
| PA0149 | PA0149 | 0.86 | STRING |
| PA3410 | PA3410 | 0.88 | STRING |
| PA1776 | *sigX* | 0.875 | STRING |
| PA2387 | *fpvI* | 0.866 | STRING |
| PA2896 | PA2896 | 0.863 | STRING |
| PA3543 | *algK* | 0.863 | STRING |
| PA1912 | *femI* | 0.86 | STRING |
| PA2093 | PA2093 | 0.86 | STRING |
| PA2050 | PA2050 | 0.86 | STRING |
| PA1363 | PA1363 | 0.86 | STRING |
| PA1300 | PA1300 | 0.86 | STRING |
| PA3546 | *algX* | 0.853 | STRING |
| PA3549 | *algJ* | 0.834 | STRING |
