# Supplementary material for: The anti-sigma factor MucA of Pseudomonas aeruginosa: Dramatic differences of a mucA22 vs. a ΔmucA mutant in anaerobic acidified nitrite sensitivity of planktonic and biofilm bacteria in vitro and during chronic murine lung infection
Source: PLoS One. 2019 Jun 3;14(6):e0216401. doi: 10.1371/journal.pone.0216401 (PMC6546240; doi:10.1371/journal.pone.0216401)
Supplement: S7 Table — (DOCX) [file pone.0216401.s009.docx]

| #pathway ID | pathway description | observed gene count | false discovery rate |
| --- | --- | --- | --- |
| GO.0016051 | carbohydrate biosynthetic process | 10 | 1.50E-07 |
| GO.0033692 | cellular polysaccharide biosynthetic process | 9 | 1.50E-07 |
| GO.0042121 | alginic acid biosynthetic process | 7 | 1.50E-07 |
| GO.0032885 | regulation of polysaccharide biosynthetic process | 3 | 7.52E-05 |
| GO.0044711 | single-organism biosynthetic process | 13 | 0.000664 |
| GO.0034645 | cellular macromolecule biosynthetic process | 11 | 0.000836 |
| GO.0044710 | single-organism metabolic process | 17 | 0.000884 |
| GO.0046394 | carboxylic acid biosynthetic process | 8 | 0.00349 |
| GO.0044699 | single-organism process | 18 | 0.00474 |
| GO.0044249 | cellular biosynthetic process | 14 | 0.0123 |
| GO.0044763 | single-organism cellular process | 16 | 0.0142 |
| GO.1901576 | organic substance biosynthetic process | 13 | 0.0394 |
